# Supplementary material for: The impacts of fine-tuning, phylogenetic distance, and sample size on big-data bioacoustics
Source: PLoS One. 2022 Dec 7;17(12):e0278522. doi: 10.1371/journal.pone.0278522 (PMC9728902; doi:10.1371/journal.pone.0278522)
Supplement: S1 File — (PDF) [file pone.0278522.s001.pdf]

Supplementary Information for: **The impacts of fine-tuning, phylogenetic distance, and sample size on big-data bioacoustics**

Short Title: Machine learning in bioacoustics

Kaiya L. Provost[1*], Jiaying Yang[1], Bryan C. Carstens[1]
1: Department of Evolution, Ecology and Organismal Biology, The Ohio State University. 318 W. 12th Ave. 300 Aronoff Laboratory Columbus, OH 43210

*: Corresponding author: Kaiya L. Provost, provost.27@osu.edu, Department of Evolution, Ecology and Organismal Biology, The Ohio State University. 318 W. 12th Ave. 300 Aronoff Laboratory Columbus, OH 43210

Supplementary Figures

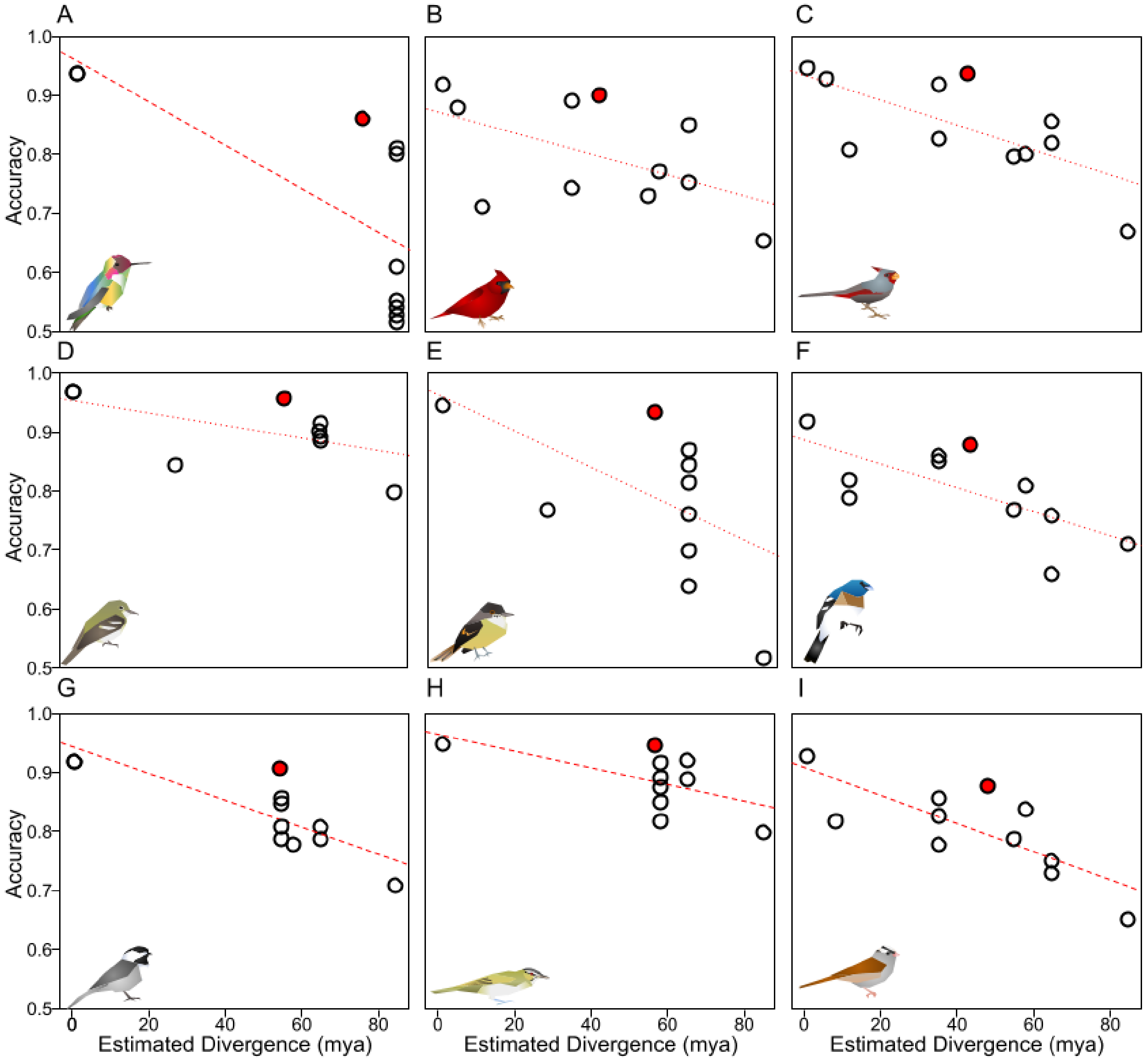

S1 Fig: Model performance by species drops with estimated divergence, but training on multiple species performs better than expected given average divergence time. X-axis gives the estimated divergence time. Y-axis gives the accuracy. Colored lines show the line of best fit, with solid lines bing significant and dotted lines not significant. Filled red points were trained on the “9 Species” model. A) *Calypte anna*. B) *Cardinalis cardinalis*. C) *Cardinalis sinuatus*. D) *Empidonax virescens*. E) *Myiarchus tuberculifer*. F) *Passerina amoena*. G) *Poecile carolinensis*. H) *Vireo altiloquus*. I) *Zonotrichia leucophrys*.

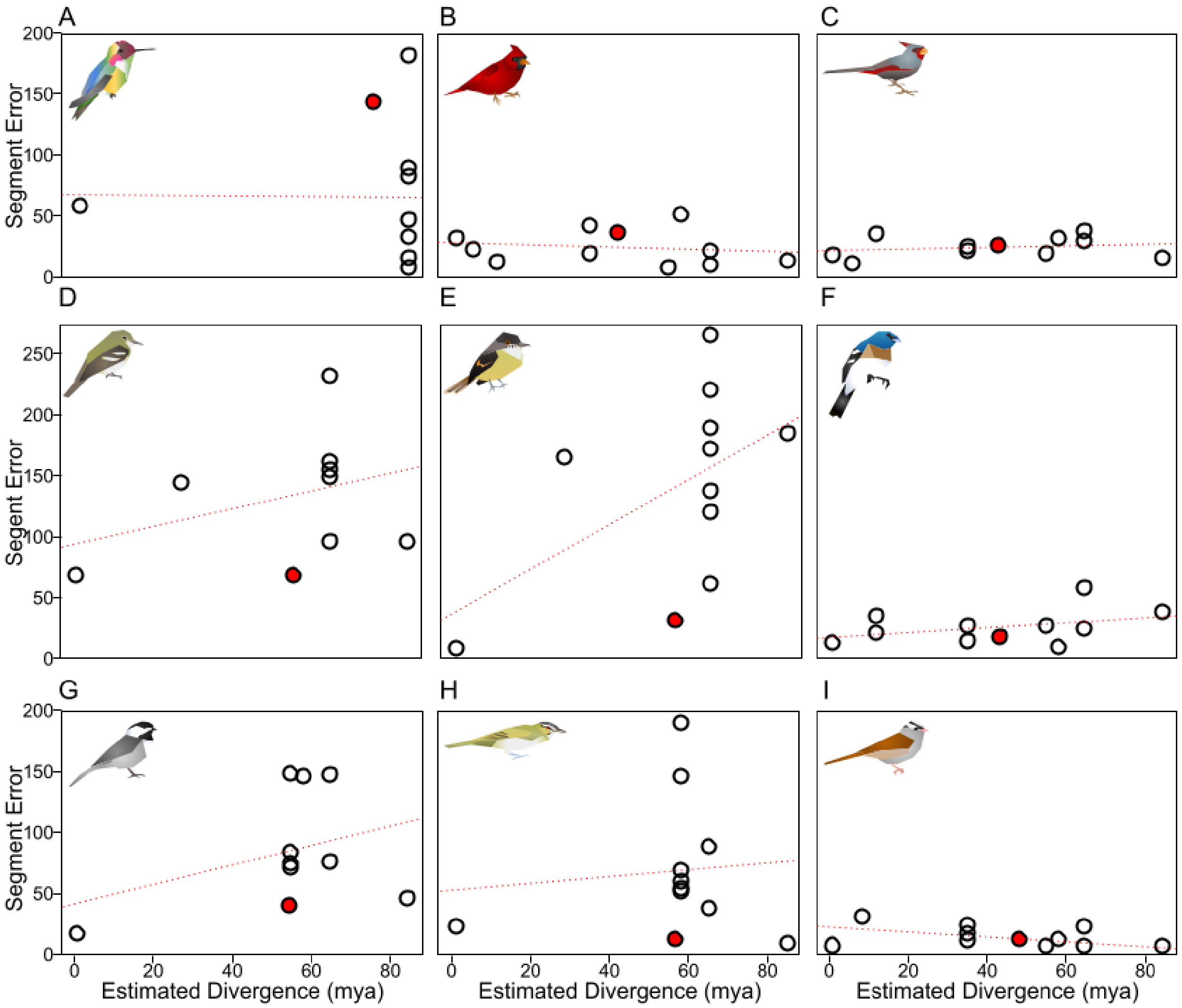

S2 Fig: Segment error rate is worse with more distant divergence times, but training on multiple species performs better than expected. X-axis gives the estimated divergence time. Y-axis gives the accuracy. Colored lines show the line of best fit, with solid lines being significant and dotted lines not significant. Filled red points were trained on the "9 Species" model. A) *Calypte anna*. B) *Cardinalis cardinalis.* C) *Cardinalis sinuatus.* D) *Empidonax virescens*. E) *Myiarchus tuberculifer*. F) *Passerina amoena.* G) *Poecile carolinensis.* H) *Vireo altiloquus.* I) *Zonotrichia leucophrys.*

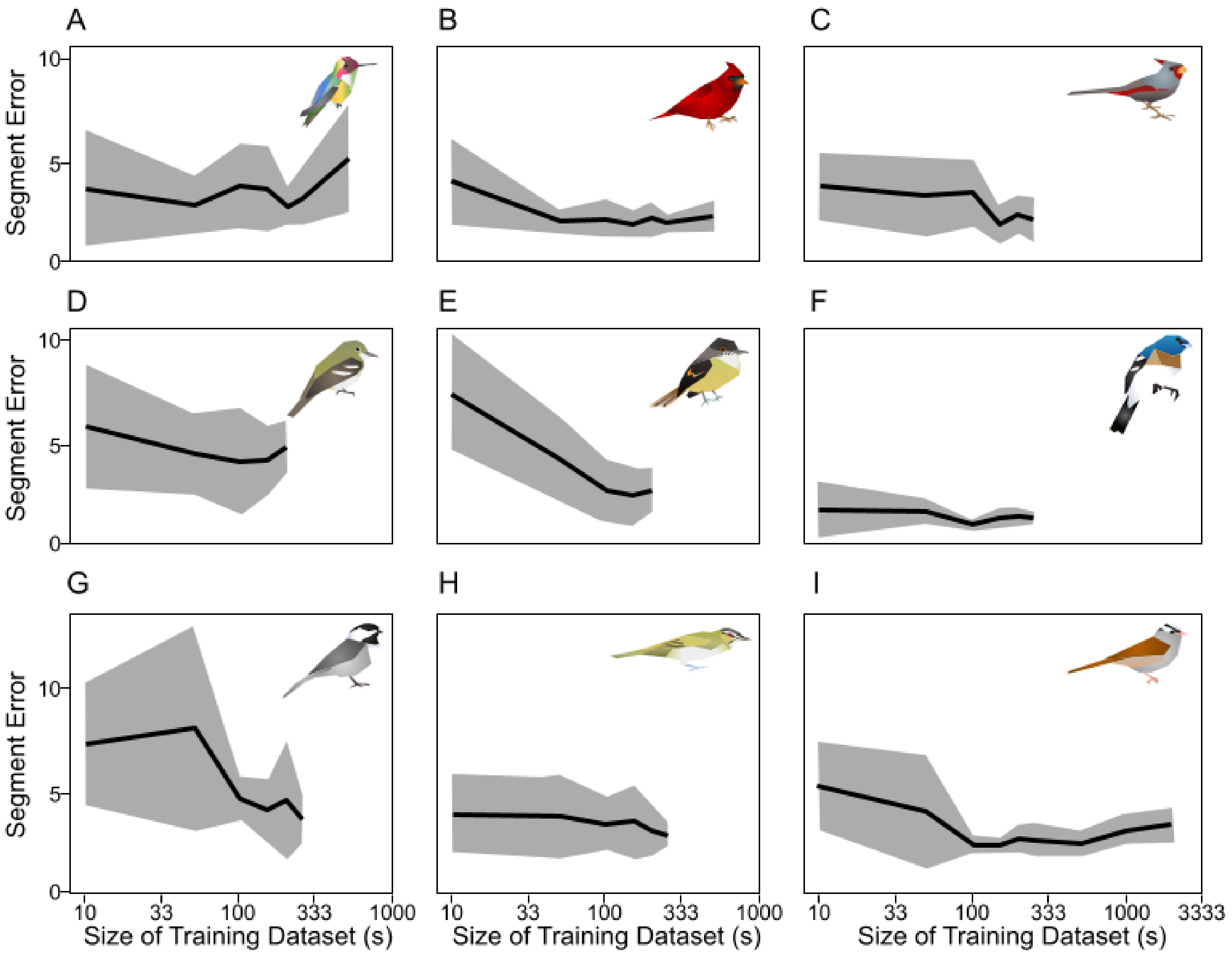

S3 Fig: Segment error rate of models improves with larger training data, but plateaus quickly. X-axis gives the size of the training dataset in seconds (log scale). Y-axis gives the uncorrected segment error rate, with higher values being worse performance. Solid line connects mean estimates across 10 replicates of data, with gray polygons giving one standard deviation. A) *Calypte anna*. B) *Cardinalis cardinalis*. C) *Cardinalis sinuatus*. D) *Empidonax virescens*. E) *Myiarchus tuberculifer*. F) *Passerina amoena*. G) *Poecile carolinensis*. H) *Vireo altiloquus*. I) *Zonotrichia leucophrys*.

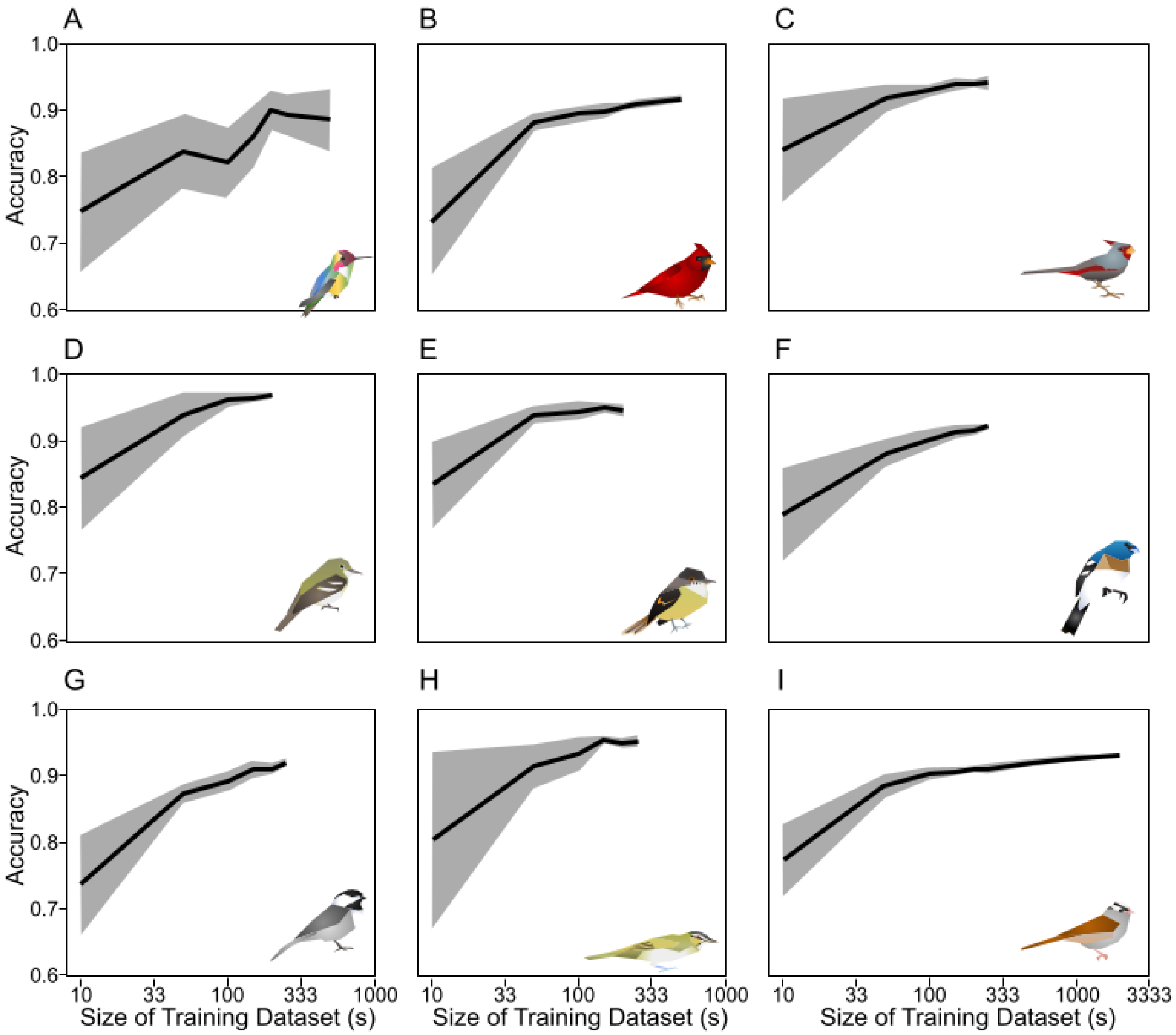

S4 Fig: Accuracy of models improves with larger training data, but plateaus quickly. X-axis gives the size of the training dataset in seconds (log scale). Y-axis gives the uncorrected accuracy, with higher values being better performance. Solid line connects mean estimates across 10 replicates of data, with gray polygons giving one standard deviation. A) *Calypte anna*. B) *Cardinalis cardinalis*. C) *Cardinalis sinuatus*. D) *Empidonax virescens*. E) *Myiarchus tuberculifer*. F) *Passerina amoena*. G) *Poecile carolinensis*. H) *Vireo altiloquus*. I) *Zonotrichia leucophrys*.

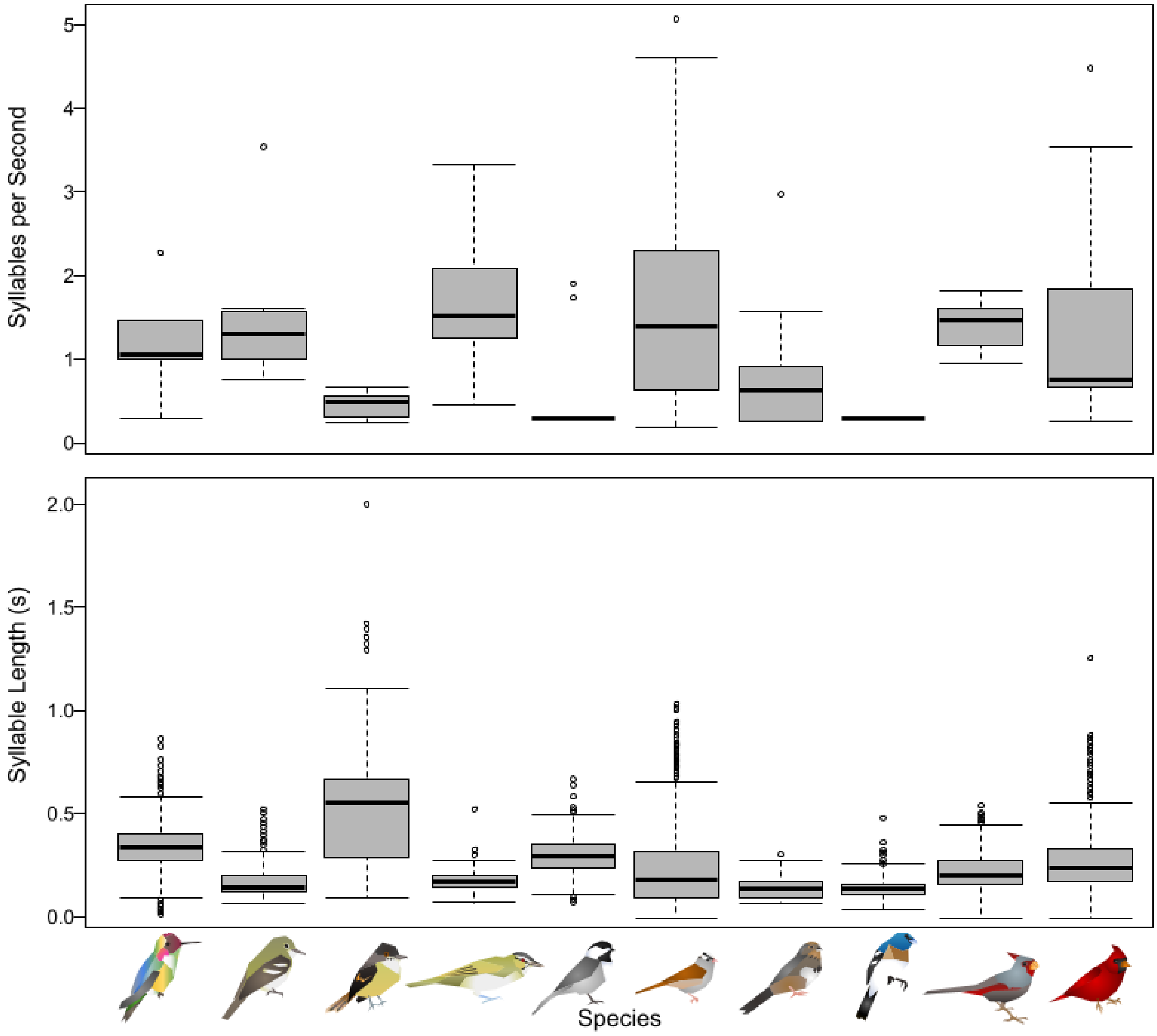

S4 Fig: Taxa vary in length of syllables as well as number of syllables per second. X-axis gives species (see Fig 2). Top: boxplot of syllables per second, aka syllable rate. Bottom: boxplot of duration of syllables in seconds.

Supplementary Tables

S1 Table: Frame Rate Accuracy and Syllable Error Rate of models trained in TweetyNet across species.

Train	Test	Acc	Prec	Rec	F	S.E.R.	Div
9 Species	*9 Species*	0.91 (0.91+0.07)	0.92 (0.90+0.16)	0.84 (0.85+0.14)	0.88 (0.86+0.14)	0.20 (0.64+1.19)	0
9 Species	*C. anna*	0.78 (0.81+0.13)	0.93 (0.79+0.36)	0.66 (0.71+0.21)	0.78 (0.68+0.29)	1.44 (2.28+2.09)	75.6
9 Species	*C. cardinalis*	0.89 (0.90+0.06)	0.95 (0.95+0.06)	0.81 (0.77+0.18)	0.87 (0.84+0.13)	0.36 (0.49+0.63)	42.1
9 Species	*C. sinuatus*	0.93 (0.93+0.05)	0.94 (0.94+0.08)	0.86 (0.87+0.14)	0.90 (0.89+0.08)	0.27 (0.35+0.36)	42.1
9 Species	*E. virescens*	0.97 (0.97+0.03)	0.87 (0.90+0.12)	0.86 (0.88+0.12)	0.87 (0.88+0.08)	0.63 (0.79+1.05)	55.7
9 Species	*M. fusca*	0.94 (0.94+0.05)	0.87 (0.79+0.26)	0.91 (0.89+0.09)	0.89 (0.81+0.20)	0.19 (1.00+1.46)	49
9 Species	*M. tuberculifer*	0.94 (0.95+0.03)	0.92 (0.95+0.07)	0.91 (0.89+0.09)	0.92 (0.91+0.05)	0.31 (0.48+0.47)	55.8
9 Species	*P. amoena*	0.87 (0.87+0.07)	0.96 (0.96+0.04)	0.72 (0.74+0.13)	0.82 (0.83+0.08)	0.09 (0.20+0.21)	42.8
9 Species	*P. carolinensis*	0.93 (0.94+0.05)	0.90 (0.89+0.15)	0.90 (0.89+0.13)	0.90 (0.88+0.11)	0.41 (0.62+0.80)	54.8
9 Species	*V. altiloquus*	0.96 (0.96+0.05)	0.97 (0.97+0.05)	0.79 (0.82+0.17)	0.87 (0.88+0.12)	0.13 (0.18+0.32)	56.1
9 Species	*Z. leucophrys*	0.90 (0.90+0.08)	0.93 (0.93+0.11)	0.84 (0.85+0.15)	0.88 (0.87+0.12)	0.13 (0.55+1.23)	48.1
C. anna	*9 Species*	0.68 (0.69+0.16)	0.69 (0.76+0.28)	0.35 (0.39+0.29)	0.46 (0.44+0.24)	0.07 (1.13+2.18)	75.6
C. anna	*C. anna*	0.90 (0.91+0.06)	0.94 (0.81+0.34)	0.88 (0.91+0.05)	0.91 (0.81+0.26)	0.58 (1.46+2.02)	0
C. anna	*C. cardinalis*	0.62 (0.65+0.17)	0.78 (0.87+0.23)	0.21 (0.27+0.19)	0.33 (0.38+0.22)	0.13 (1.12+1.91)	85
C. anna	*C. sinuatus*	0.65 (0.66+0.08)	0.64 (0.76+0.32)	0.15 (0.17+0.18)	0.25 (0.24+0.21)	0.16 (0.78+0.85)	85
C. anna	*E. virescens*	0.81 (0.81+0.23)	0.33 (0.71+0.35)	0.52 (0.54+0.36)	0.40 (0.45+0.23)	0.89 (1.49+1.93)	85
C. anna	*M. fusca*	0.80 (0.82+0.15)	0.65 (0.69+0.32)	0.37 (0.46+0.31)	0.47 (0.49+0.28)	0.18 (1.50+2.93)	85
C. anna	*M. tuberculifer*	0.56 (0.56+0.21)	0.37 (0.42+0.24)	0.35 (0.54+0.35)	0.36 (0.37+0.22)	1.86 (5.93+7.11)	85
C. anna	*P. amoena*	0.69 (0.70+0.10)	0.91 (0.93+0.06)	0.31 (0.33+0.24)	0.46 (0.44+0.23)	0.30 (0.41+0.21)	85
C. anna	*P. carolinensis*	0.72 (0.74+0.21)	0.56 (0.65+0.31)	0.72 (0.68+0.32)	0.63 (0.65+0.16)	0.46 (0.94+0.72)	85
C. anna	*V. altiloquus*	0.82 (0.82+0.04)	0.40 (0.30+0.27)	0.19 (0.18+0.20)	0.26 (0.33+0.18)	0.10 (1.12+0.62)	85
C. anna	*Z. leucophrys*	0.64 (0.64+0.12)	0.70 (0.80+0.23)	0.33 (0.35+0.25)	0.45 (0.42+0.21)	0.02 (0.76+0.95)	85
C. cardinalis	*9 Species*	0.85 (0.86+0.12)	0.87 (0.85+0.21)	0.73 (0.77+0.23)	0.79 (0.77+0.20)	0.26 (0.96+1.56)	42.1
C. cardinalis	*C. anna*	0.40 (0.48+0.13)	0.43 (0.35+0.42)	0.07 (0.09+0.08)	0.12 (0.08+0.07)	0.33 (2.19+3.25)	85
C. cardinalis	*C. cardinalis*	0.92 (0.92+0.05)	0.95 (0.96+0.05)	0.86 (0.82+0.18)	0.90 (0.87+0.13)	0.31 (0.37+0.49)	0

C. cardinalis	*C. sinuatus*	0.92 (0.92+0.06)	0.92 (0.92+0.09)	0.87 (0.88+0.18)	0.89 (0.88+0.12)	0.11 (0.23+0.19)	5
C. cardinalis	*E. virescens*	0.90 (0.91+0.09)	0.57 (0.72+0.28)	0.91 (0.91+0.10)	0.70 (0.76+0.19)	2.11 (1.82+1.76)	65
C. cardinalis	*M. fusca*	0.92 (0.91+0.05)	0.77 (0.69+0.27)	0.95 (0.93+0.08)	0.85 (0.76+0.21)	0.10 (1.38+1.85)	35
C. cardinalis	*M. tuberculifer*	0.87 (0.88+0.07)	0.81 (0.88+0.18)	0.83 (0.78+0.21)	0.82 (0.79+0.12)	1.90 (2.99+3.23)	65
C. cardinalis	*P. amoena*	0.76 (0.77+0.12)	0.95 (0.96+0.05)	0.48 (0.52+0.22)	0.63 (0.64+0.19)	0.12 (0.21+0.25)	11
C. cardinalis	*P. carolinensis*	0.82 (0.84+0.17)	0.80 (0.76+0.31)	0.63 (0.67+0.34)	0.70 (0.74+0.24)	0.74 (0.99+0.93)	55
C. cardinalis	*V. altiloquus*	0.85 (0.86+0.16)	0.57 (0.73+0.26)	0.88 (0.89+0.14)	0.69 (0.77+0.19)	1.90 (1.62+2.38)	58
C. cardinalis	*Z. leucophrys*	0.84 (0.84+0.11)	0.90 (0.91+0.13)	0.71 (0.71+0.22)	0.80 (0.77+0.19)	0.24 (0.71+1.16)	35
C. sinuatus	*9 Species*	0.83 (0.84+0.13)	0.83 (0.83+0.20)	0.71 (0.75+0.24)	0.77 (0.74+0.20)	0.24 (0.78+1.26)	42.1
C. sinuatus	*C. anna*	0.67 (0.75+0.20)	0.91 (0.71+0.37)	0.47 (0.64+0.38)	0.62 (0.57+0.37)	0.33 (1.24+1.56)	85
C. sinuatus	*C. cardinalis*	0.88 (0.88+0.09)	0.91 (0.93+0.08)	0.81 (0.77+0.25)	0.86 (0.81+0.21)	0.22 (0.44+0.70)	5
C. sinuatus	*C. sinuatus*	0.95 (0.95+0.03)	0.94 (0.93+0.08)	0.92 (0.93+0.07)	0.93 (0.92+0.05)	0.19 (0.27+0.29)	0
C. sinuatus	*E. virescens*	0.93 (0.93+0.06)	0.67 (0.71+0.24)	0.90 (0.90+0.09)	0.76 (0.78+0.16)	1.42 (1.33+1.48)	65
C. sinuatus	*M. fusca*	0.93 (0.93+0.05)	0.83 (0.76+0.27)	0.89 (0.89+0.08)	0.86 (0.79+0.20)	0.23 (1.03+1.95)	35
C. sinuatus	*M. tuberculifer*	0.69 (0.71+0.29)	0.54 (0.70+0.31)	0.89 (0.86+0.16)	0.67 (0.71+0.20)	0.62 (1.37+1.56)	65
C. sinuatus	*P. amoena*	0.81 (0.81+0.08)	0.85 (0.87+0.11)	0.68 (0.68+0.21)	0.76 (0.74+0.11)	0.27 (0.33+0.31)	11
C. sinuatus	*P. carolinensis*	0.83 (0.84+0.13)	0.70 (0.74+0.17)	0.88 (0.88+0.13)	0.78 (0.79+0.13)	0.83 (0.95+0.81)	55
C. sinuatus	*V. altiloquus*	0.83 (0.84+0.16)	0.53 (0.68+0.30)	0.81 (0.85+0.18)	0.64 (0.71+0.21)	1.47 (1.49+1.65)	58
C. sinuatus	*Z. leucophrys*	0.79 (0.80+0.12)	0.85 (0.87+0.14)	0.65 (0.65+0.25)	0.74 (0.71+0.21)	0.21 (0.64+0.95)	35
E. virescens	*9 Species*	0.77 (0.79+0.13)	0.92 (0.91+0.18)	0.45 (0.52+0.27)	0.60 (0.61+0.23)	0.25 (0.81+1.25)	55.7
E. virescens	*C. anna*	0.41 (0.55+0.23)	0.77 (0.48+0.49)	0.09 (0.04+0.06)	0.17 (0.12+0.11)	0.47 (1.23+1.02)	85
E. virescens	*C. cardinalis*	0.70 (0.72+0.15)	0.99 (0.96+0.16)	0.34 (0.37+0.23)	0.51 (0.50+0.26)	0.20 (0.47+0.46)	65
E. virescens	*C. sinuatus*	0.80 (0.80+0.04)	0.97 (0.97+0.03)	0.47 (0.46+0.12)	0.63 (0.61+0.12)	0.38 (0.53+0.35)	65
E. virescens	*E. virescens*	0.97 (0.97+0.03)	0.97 (0.97+0.05)	0.79 (0.80+0.16)	0.87 (0.87+0.11)	0.63 (0.63+0.71)	0
E. virescens	*M. fusca*	0.91 (0.91+0.06)	0.86 (0.80+0.26)	0.77 (0.79+0.19)	0.81 (0.75+0.19)	0.18 (0.96+1.17)	65
E. virescens	*M. tuberculifer*	0.79 (0.82+0.14)	0.85 (0.89+0.15)	0.53 (0.63+0.24)	0.65 (0.70+0.13)	1.66 (3.42+3.73)	27
E. virescens	*P. amoena*	0.73 (0.74+0.13)	0.92 (0.95+0.07)	0.39 (0.44+0.22)	0.55 (0.56+0.26)	0.15 (0.35+0.27)	65

E. virescens	*P. carolinensis*	0.83 (0.84+0.12)	0.83 (0.87+0.20)	0.61 (0.60+0.21)	0.71 (0.69+0.17)	1.48 (1.49+1.76)	65
E. virescens	*V. altiloquus*	0.92 (0.92+0.07)	0.93 (0.93+0.09)	0.62 (0.65+0.23)	0.75 (0.74+0.19)	0.40 (0.60+0.43)	65
E. virescens	*Z. leucophrys*	0.73 (0.73+0.11)	0.94 (0.95+0.14)	0.42 (0.41+0.22)	0.58 (0.54+0.22)	0.25 (0.65+0.95)	65
9 Species+M. fusca	*9 Species*	0.89 (0.90+0.10)	0.88 (0.90+0.14)	0.82 (0.84+0.18)	0.85 (0.85+0.15)	0.36 (0.73+1.23)	49
9 Species+M. fusca	*C. anna*	0.74 (0.79+0.16)	0.91 (0.73+0.23)	0.61 (0.67+0.33)	0.73 (0.63+0.28)	1.81 (2.23+1.32)	85
9 Species+M. fusca	*C. cardinalis*	0.88 (0.89+0.06)	0.94 (0.95+0.07)	0.79 (0.79+0.15)	0.86 (0.85+0.10)	0.42 (0.64+1.08)	35
9 Species+M. fusca	*C. sinuatus*	0.91 (0.91+0.06)	0.96 (0.96+0.07)	0.78 (0.79+0.18)	0.86 (0.85+0.13)	0.22 (0.34+0.31)	35
9 Species+M. fusca	*E. virescens*	0.95 (0.95+0.04)	0.75 (0.81+0.17)	0.88 (0.88+0.10)	0.81 (0.82+0.09)	1.37 (1.25+1.14)	65
9 Species+M. fusca	*M. fusca*	0.98 (0.98+0.02)	0.97 (0.96+0.09)	0.95 (0.93+0.08)	0.96 (0.94+0.07)	0.08 (0.18+0.53)	0
9 Species+M. fusca	*M. tuberculifer*	0.88 (0.90+0.08)	0.83 (0.91+0.12)	0.85 (0.80+0.21)	0.84 (0.83+0.14)	1.72 (2.73+3.23)	65
9 Species+M. fusca	*P. amoena*	0.85 (0.84+0.07)	0.89 (0.90+0.10)	0.75 (0.73+0.19)	0.81 (0.79+0.11)	0.05 (0.35+0.26)	35
9 Species+M. fusca	*P. carolinensis*	0.91 (0.91+0.05)	0.88 (0.87+0.16)	0.86 (0.87+0.13)	0.87 (0.85+0.11)	0.72 (0.98+0.89)	55
9 Species+M. fusca	*V. altiloquus*	0.90 (0.90+0.11)	0.69 (0.81+0.20)	0.86 (0.85+0.24)	0.76 (0.78+0.21)	0.70 (0.79+0.83)	58
9 Species+M. fusca	*Z. leucophrys*	0.86 (0.87+0.11)	0.87 (0.88+0.14)	0.82 (0.82+0.19)	0.84 (0.83+0.16)	0.32 (0.76+1.19)	8
M. tuberculifer	*9 Species*	0.78 (0.79+0.12)	0.89 (0.87+0.23)	0.50 (0.54+0.25)	0.64 (0.62+0.22)	0.03 (0.66+0.94)	55.8
M. tuberculifer	*C. anna*	0.37 (0.49+0.21)	0.44 (0.49+0.47)	0.03 (0.02+0.02)	0.06 (0.06+0.01)	0.82 (0.92+0.11)	85
M. tuberculifer	*C. cardinalis*	0.84 (0.85+0.08)	0.95 (0.95+0.06)	0.69 (0.71+0.16)	0.80 (0.80+0.11)	0.09 (0.40+0.35)	65
M. tuberculifer	*C. sinuatus*	0.85 (0.85+0.05)	0.94 (0.95+0.07)	0.63 (0.66+0.16)	0.75 (0.76+0.10)	0.30 (0.28+0.21)	65
M. tuberculifer	*E. virescens*	0.85 (0.85+0.19)	0.45 (0.72+0.30)	0.64 (0.65+0.25)	0.53 (0.61+0.23)	1.32 (1.24+0.97)	27
M. tuberculifer	*M. fusca*	0.88 (0.88+0.06)	0.82 (0.73+0.32)	0.66 (0.65+0.20)	0.73 (0.66+0.22)	0.22 (1.05+1.52)	65
M. tuberculifer	*M. tuberculifer*	0.95 (0.95+0.04)	0.93 (0.96+0.06)	0.92 (0.92+0.10)	0.92 (0.93+0.06)	0.03 (0.18+0.32)	0
M. tuberculifer	*P. amoena*	0.64 (0.65+0.10)	0.97 (0.97+0.06)	0.16 (0.19+0.12)	0.27 (0.30+0.16)	0.51 (0.54+0.14)	65
M. tuberculifer	*P. carolinensis*	0.80 (0.81+0.10)	0.83 (0.81+0.25)	0.54 (0.54+0.25)	0.66 (0.62+0.24)	0.76 (0.91+0.77)	65
M. tuberculifer	*V. altiloquus*	0.89 (0.90+0.11)	0.67 (0.78+0.21)	0.81 (0.83+0.12)	0.73 (0.78+0.13)	0.90 (0.74+0.95)	65
M. tuberculifer	*Z. leucophrys*	0.74 (0.74+0.10)	0.93 (0.92+0.18)	0.45 (0.44+0.21)	0.60 (0.58+0.20)	0.02 (0.55+0.74)	65
P. amoena	*9 Species*	0.85 (0.87+0.12)	0.87 (0.86+0.20)	0.73 (0.77+0.21)	0.80 (0.78+0.20)	0.25 (0.82+1.48)	42.8
P. amoena	*C. anna*	0.68 (0.72+0.20)	0.88 (0.67+0.43)	0.51 (0.58+0.33)	0.64 (0.54+0.41)	0.88 (1.45+1.45)	85

P. amoena	*C. cardinalis*	0.68 (0.72+0.16)	0.87 (0.90+0.16)	0.35 (0.42+0.21)	0.50 (0.54+0.23)	0.12 (0.77+0.81)	11
P. amoena	*C. sinuatus*	0.79 (0.79+0.05)	0.92 (0.91+0.11)	0.47 (0.46+0.13)	0.62 (0.61+0.13)	0.36 (0.40+0.33)	11
P. amoena	*E. virescens*	0.94 (0.94+0.04)	0.71 (0.75+0.20)	0.87 (0.87+0.10)	0.78 (0.79+0.12)	1.47 (1.72+1.77)	65
P. amoena	*M. fusca*	0.93 (0.93+0.05)	0.84 (0.78+0.29)	0.89 (0.90+0.11)	0.87 (0.79+0.22)	0.32 (0.99+1.43)	35
P. amoena	*M. tuberculifer*	0.64 (0.64+0.27)	0.51 (0.62+0.27)	0.60 (0.62+0.31)	0.55 (0.52+0.26)	2.66 (4.35+4.22)	65
P. amoena	*P. amoena*	0.93 (0.93+0.05)	0.95 (0.94+0.04)	0.87 (0.88+0.08)	0.91 (0.91+0.05)	0.04 (0.12+0.10)	0
P. amoena	*P. carolinensis*	0.87 (0.88+0.09)	0.77 (0.75+0.18)	0.88 (0.85+0.22)	0.82 (0.79+0.19)	0.83 (0.89+0.68)	55
P. amoena	*V. altiloquus*	0.90 (0.90+0.06)	0.85 (0.91+0.15)	0.52 (0.53+0.23)	0.65 (0.64+0.21)	0.57 (0.56+0.70)	58
P. amoena	*Z. leucophrys*	0.87 (0.87+0.09)	0.90 (0.91+0.12)	0.79 (0.79+0.16)	0.84 (0.83+0.12)	0.14 (0.56+1.09)	35
P. carolinensis	*9 Species*	0.80 (0.82+0.12)	0.95 (0.93+0.16)	0.52 (0.55+0.22)	0.67 (0.66+0.20)	0.10 (0.61+1.25)	54.8
P. carolinensis	*C. anna*	0.50 (0.60+0.16)	0.80 (0.68+0.45)	0.14 (0.16+0.14)	0.24 (0.19+0.19)	0.16 (1.13+1.65)	85
P. carolinensis	*C. cardinalis*	0.70 (0.72+0.15)	0.97 (0.96+0.13)	0.35 (0.37+0.25)	0.51 (0.50+0.27)	0.08 (0.75+0.61)	55
P. carolinensis	*C. sinuatus*	0.77 (0.77+0.04)	0.97 (0.96+0.07)	0.40 (0.39+0.10)	0.56 (0.55+0.11)	0.20 (0.31+0.30)	55
P. carolinensis	*E. virescens*	0.90 (0.90+0.14)	0.62 (0.87+0.25)	0.61 (0.61+0.23)	0.61 (0.66+0.20)	1.37 (1.22+2.22)	65
P. carolinensis	*M. fusca*	0.91 (0.92+0.06)	0.96 (0.88+0.23)	0.68 (0.62+0.23)	0.80 (0.71+0.20)	0.24 (0.59+0.74)	55
P. carolinensis	*M. tuberculifer*	0.80 (0.83+0.13)	0.86 (0.83+0.18)	0.53 (0.60+0.23)	0.66 (0.68+0.18)	1.38 (2.21+2.42)	65
P. carolinensis	*P. amoena*	0.75 (0.76+0.11)	0.94 (0.95+0.06)	0.44 (0.48+0.20)	0.60 (0.61+0.16)	0.18 (0.33+0.29)	55
P. carolinensis	*P. carolinensis*	0.95 (0.95+0.04)	0.95 (0.93+0.14)	0.90 (0.88+0.12)	0.92 (0.89+0.11)	0.17 (0.30+0.57)	0
P. carolinensis	*V. altiloquus*	0.88 (0.88+0.07)	0.78 (0.90+0.18)	0.50 (0.49+0.25)	0.61 (0.59+0.22)	0.63 (0.83+0.81)	58
P. carolinensis	*Z. leucophrys*	0.78 (0.79+0.10)	0.96 (0.96+0.10)	0.54 (0.55+0.18)	0.69 (0.68+0.16)	0.04 (0.49+1.32)	55
V. altiloquus	*9 Species*	0.83 (0.84+0.13)	0.86 (0.85+0.24)	0.70 (0.73+0.25)	0.77 (0.75+0.21)	0.27 (1.05+1.98)	56.1
V. altiloquus	*C. anna*	0.37 (0.41+0.13)	0.44 (0.30+0.47)	0.10 (0.28+0.40)	0.17 (0.15+0.06)	0.02 (2.45+4.25)	85
V. altiloquus	*C. cardinalis*	0.74 (0.76+0.12)	0.91 (0.92+0.12)	0.51 (0.55+0.15)	0.65 (0.67+0.13)	0.50 (1.02+1.33)	58
V. altiloquus	*C. sinuatus*	0.80 (0.79+0.07)	0.87 (0.87+0.19)	0.54 (0.54+0.14)	0.67 (0.65+0.14)	0.33 (0.44+0.65)	58
V. altiloquus	*E. virescens*	0.90 (0.90+0.20)	0.59 (0.81+0.23)	0.81 (0.81+0.20)	0.69 (0.77+0.21)	1.42 (1.42+2.37)	65
V. altiloquus	*M. fusca*	0.87 (0.85+0.15)	0.69 (0.63+0.38)	0.86 (0.78+0.29)	0.77 (0.70+0.29)	0.55 (2.19+3.32)	58
V. altiloquus	*M. tuberculifer*	0.90 (0.91+0.05)	0.86 (0.87+0.10)	0.88 (0.87+0.13)	0.87 (0.86+0.08)	1.21 (2.03+2.02)	65

V. altiloquus	*P. amoena*	0.79 (0.80+0.08)	0.88 (0.89+0.10)	0.60 (0.62+0.19)	0.71 (0.71+0.15)	0.00 (0.22+0.16)	58
V. altiloquus	*P. carolinensis*	0.82 (0.84+0.13)	0.70 (0.78+0.20)	0.82 (0.83+0.12)	0.76 (0.78+0.11)	1.46 (1.55+1.06)	58
V. altiloquus	*V. altiloquus*	0.95 (0.95+0.04)	0.89 (0.93+0.09)	0.84 (0.85+0.15)	0.87 (0.87+0.10)	0.23 (0.22+0.33)	0
V. altiloquus	*Z. leucophrys*	0.85 (0.85+0.12)	0.92 (0.93+0.12)	0.73 (0.72+0.24)	0.81 (0.78+0.20)	0.12 (0.63+1.18)	58
Z. leucophrys	*9 Species*	0.88 (0.90+0.11)	0.91 (0.89+0.20)	0.78 (0.78+0.24)	0.84 (0.80+0.21)	0.07 (0.73+1.97)	48.1
Z. leucophrys	*C. anna*	0.39 (0.46+0.11)	0.58 (0.43+0.49)	0.10 (0.05+0.06)	0.17 (0.12+0.11)	0.33 (1.80+2.37)	85
Z. leucophrys	*C. cardinalis*	0.69 (0.71+0.13)	0.94 (0.94+0.10)	0.35 (0.39+0.19)	0.51 (0.53+0.20)	0.18 (0.67+0.54)	35
Z. leucophrys	*C. sinuatus*	0.81 (0.81+0.06)	0.93 (0.93+0.13)	0.53 (0.52+0.13)	0.67 (0.65+0.12)	0.25 (0.40+0.36)	35
Z. leucophrys	*E. virescens*	0.91 (0.92+0.06)	0.63 (0.77+0.26)	0.81 (0.80+0.25)	0.71 (0.72+0.22)	0.89 (1.00+0.80)	65
Z. leucophrys	*M. fusca*	0.92 (0.92+0.05)	0.86 (0.77+0.32)	0.82 (0.68+0.24)	0.84 (0.69+0.28)	0.28 (0.98+1.65)	8
Z. leucophrys	*M. tuberculifer*	0.76 (0.76+0.18)	0.68 (0.78+0.29)	0.64 (0.67+0.19)	0.66 (0.66+0.14)	2.21 (5.24+9.14)	65
Z. leucophrys	*P. amoena*	0.83 (0.83+0.07)	0.91 (0.92+0.08)	0.66 (0.68+0.16)	0.77 (0.77+0.09)	0.19 (0.25+0.20)	35
Z. leucophrys	*P. carolinensis*	0.82 (0.83+0.15)	0.71 (0.78+0.21)	0.80 (0.79+0.11)	0.75 (0.77+0.12)	1.48 (1.47+1.53)	55
Z. leucophrys	*V. altiloquus*	0.93 (0.93+0.06)	0.81 (0.89+0.15)	0.78 (0.76+0.23)	0.79 (0.79+0.16)	0.60 (0.53+0.67)	58
Z. leucophrys	*Z. leucophrys*	0.94 (0.94+0.05)	0.95 (0.95+0.08)	0.91 (0.91+0.13)	0.93 (0.92+0.10)	0.08 (0.34+0.43)	0

Numerical estimates for performance (Acc, Prec, Rec, F, S.E.R) are given as point estimate (mean+standard deviation). “Train” = species model was trained on. “Test” = species model was tested on. “Acc” = model accuracy. “Prec” = model precision. “Rec” = model recall. “F” = model F-score. “S.E.R” = absolute model segment error rate. “Div” = divergence time between trained and tested species. Estimated divergence time for “9 Species” models is a weighted average, proportional to the sample sizes used for each species trained on.

S2 Table: Statistical significance and model parameters of ANOVA tests.

Response	Predictor	F	p	Sum squares	Mean squares
accuracy	TEST	10.74	**1.57×10^{-12}**	1.00	0.10
accuracy	TRAIN	1.92	*0.049*	0.30	0.030
accuracy (mean)	TEST	8.11	**1.03×10^{-9}**	0.66	0.066
accuracy (mean)	TRAIN	2.37	*0.013*	0.27	0.027
F-score	TEST	3.42	*0.00061*	1.10	0.11
F-score	TRAIN	5.05	**4.84×10^{-6}**	1.46	0.14
F-score (mean)	TEST	4.81	**9.84×10^{-6}**	1.36	0.13
F-score (mean)	TRAIN	4.57	**1.98×10^{-5}**	1.31	0.13
precision	TEST	5.59	**1.03×10^{-6}**	0.97	0.097
precision	TRAIN	3.62	**0.00033**	0.71	0.071
precision (mean)	TEST	9.81	**1.41×10^{-11}**	1.14	0.11
precision (mean)	TRAIN	2.14	*0.026*	0.39	0.039
recall	TEST	4.79	**1.04×10^{-5}**	2.01	0.20
recall	TRAIN	5.39	**1.80×10^{-6}**	2.18	0.21
recall (mean)	TEST	4.18	**6.30×10^{-5}**	1.64	0.16
recall (mean)	TRAIN	5.44	**1.59×10^{-6}**	1.97	0.19
S.E.R.	TEST	14.2	**7.62×10^{-16}**	225828	22583
S.E.R.	TRAIN	0.48	0.89	16952	1695
S.E.R. (mean)	TEST	13.3	**5.10×10^{-15}**	551384	55138
S.E.R. (mean)	TRAIN	0.85	0.57	72857	7286
Uncorrected accuracy	TEST	5.52	**1.25×10^{-6}**	0.41	0.041
Uncorrected accuracy	TRAIN	2.92	*0.0027*	0.26	0.026
Uncorrected S.E.R.	TEST	13.39	**4.19×10^{-15}**	4288	428.80
Uncorrected S.E.R.	TRAIN	2.18	*0.024*	1292	129.18

Degrees of freedom for all analyses in this table is 110. “S.E.R” = absolute model segment error rate.
“italics” = near significant ($p < 0.05$)
“bold” = significant ($p < 0.000413$)

S3 Table: Statistical significance and model parameters of linear models between species' mean performance and song diversity.

Response	Predictor	F	Coef.	Err.	T	p	R^2	aR^2
accuracy	Test Complexity	2.05	0.016	0.011	1.43	0.21	0.29	0.14
accuracy	Test Hypervolume (mean)	0.27	0.062	0.11	0.52	0.62	0.051	-0.13
accuracy	Test Hypervolume (sd)	0.13	0.043	0.11	0.36	0.72	0.026	-0.16
accuracy	Test InVar	0.010	-0.0023	0.023	-0.10	0.92	0.0020	-0.19
accuracy	Test SpVar	2.54	-0.021	0.013	-1.59	0.17	0.33	0.20
accuracy	Train Complexity	8.50	-0.011	0.0037	-2.91	*0.033*	0.62	0.55
accuracy	Train Hypervolume (mean)	1.32	-0.056	0.048	-1.15	0.30	0.21	0.052
accuracy	Train Hypervolume (sd)	2.97	-0.073	0.042	-1.72	0.14	0.37	0.24
accuracy	Train InVar	3.06	0.014	0.0081	1.75	0.14	0.38	0.25
accuracy	Train SpVar	2.07	0.0090	0.0062	1.44	0.20	0.29	0.15
F-score	Test Complexity	0.60	0.010	0.013	0.78	0.47	0.10	-0.069
F-score	Test Hypervolume (mean)	12.94	0.23	0.064	3.59	*0.015*	0.72	0.66
F-score	Test Hypervolume (sd)	24.77	0.24	0.048	4.97	*0.0041*	0.83	0.79
F-score	Test InVar	2.15	-0.028	0.019	-1.46	0.20	0.30	0.16
F-score	Test SpVar	0.69	-0.012	0.015	-0.83	0.44	0.12	-0.053
F-score	Train Complexity	3.67	-0.027	0.014	-1.91	0.11	0.42	0.30
F-score	Train Hypervolume (mean)	2.88	-0.22	0.13	-1.69	0.15	0.36	0.23
F-score	Train Hypervolume (sd)	5.60	-0.26	0.11	-2.36	0.064	0.52	0.43
F-score	Train InVar	5.15	0.050	0.022	2.27	0.072	0.50	0.40
F-score	Train SpVar	1.38	0.023	0.020	1.17	0.29	0.21	0.060
precision	Test Complexity	29.57	-0.061	0.011	-5.43	*0.0028*	0.85	0.82
precision	Test Hypervolume (mean)	0.47	-0.17	0.24	-0.69	0.52	0.087	-0.095
precision	Test Hypervolume (sd)	0.78	-0.21	0.23	-0.88	0.417	0.13	-0.037
precision	Test InVar	0.87	0.042	0.045	0.93	0.39	0.14	-0.021
precision	Test SpVar	4.81	0.055	0.025	2.19	0.079	0.49	0.38
precision	Train Complexity	0.46	0.0087	0.012	0.68	0.52	0.085	-0.097
precision	Train Hypervolume (mean)	28.72	0.24	0.045	5.36	*0.0030*	0.85	0.82
precision	Train Hypervolume (sd)	16.57	0.22	0.055	4.07	*0.0096*	0.76	0.72
precision	Train InVar	8.85	-0.039	0.013	-2.97	*0.031*	0.63	0.56
precision	Train SpVar	2.28	-0.019	0.013	-1.51	0.19	0.31	0.17
recall	Test Complexity	5.83	0.048	0.020	2.41	0.060	0.53	0.44
recall	Test Hypervolume (mean)	5.15	0.41	0.18	2.27	0.072	0.50	0.40
recall	Test Hypervolume (sd)	7.65	0.44	0.16	2.76	*0.039*	0.60	0.52
recall	Test InVar	2.46	-0.063	0.040	-1.57	0.17	0.33	0.19
recall	Test SpVar	3.28	-0.049	0.027	-1.81	0.13	0.39	0.27
recall	Train Complexity	3.88	-0.047	0.024	-1.97	0.10	0.43	0.32
recall	Train Hypervolume (mean)	6.20	-0.47	0.19	-2.49	0.055	0.55	0.46
recall	Train Hypervolume (sd)	11.12	-0.52	0.15	-3.33	*0.020*	0.68	0.62
recall	Train InVar	12.20	0.10	0.029	3.49	*0.017*	0.70	0.65
recall	Train SpVar	2.87	0.052	0.030	1.69	0.15	0.36	0.23
S.E.R.	Test Complexity	8.79	29.00	9.78	2.96	*0.031*	0.63	0.56
S.E.R.	Test Hypervolume (mean)	0.36	83.39	138.64	0.60	0.57	0.067	-0.11
S.E.R.	Test Hypervolume (sd)	0.77	114.84	130.73	0.87	0.42	0.13	-0.039
S.E.R.	Test InVar	0.54	-18.89	25.70	-0.73	0.49	0.097	-0.08
S.E.R.	Test SpVar	1.58	-21.29	16.89	-1.26	0.26	0.24	0.089
S.E.R.	Train Complexity	4.02	-6.01	2.99	-2.00	0.10	0.44	0.33
S.E.R.	Train Hypervolume (mean)	3.01	-48.77	28.09	-1.73	0.14	0.37	0.25
S.E.R.	Train Hypervolume (sd)	4.05	-52.05	25.86	-2.01	0.10	0.44	0.33
S.E.R.	Train InVar	0.60	4.91	6.33	0.77	0.47	0.10	-0.070
S.E.R.	Train SpVar	2.29	6.02	3.97	1.51	0.19	0.31	0.17

Degrees of freedom for all analyses in this table is 5. “S.E.R” = absolute model segment error rate. “Sd” = standard deviation. “InVar” = individual variation. “SpVar” = species variation. Note that Complexity, InVar, and SpVar are taken from Medina and Francis 2012 [Ref 68]. “Coef” = coefficient value. “Err” = standard error of coefficient. “aR^2” = adjusted R^2 value.
“italics” = near significant ($p < 0.05$)
“bold” = significant ($p < 0.000413$)

S4 Table: Calculated hypervolumes correlate highly with pseudo hypervolumes at high levels of dimensionality.

**Dimensions**	**F**	**Coef.**	**Err.**	**T**	**p**	**R^2**	**aR^2**
2	0.17	1.68×10^{-3}	0.0040	0.41	0.68	0.0035	-0.017
3	4.24	2.32×10^{-3}	0.0011	2.06	*0.044*	0.081	0.062
4	13.67	8.98×10^{-4}	0.00024	3.69	*0.00055*	0.22	0.20
5	30.73	3.41×10^{-4}	0.000061	5.54	**1.24×10^{-6}**	0.39	0.37
6	57.95	1.29×10^{-4}	0.000016	7.61	**8.52×10^{-10}**	0.54	0.53
7	72.88	4.99×10^{-5}	0.0000058	8.53	**3.44×10^{-11}**	0.60	0.59

Degrees of freedom for all analyses in this table is 48. "Coef" = coefficient value. "Err" = standard error of coefficient. "aR^2" = adjusted R^2 value.
"italics" = near significant ($p < 0.05$)
"bold" = significant ($p < 0.000413$)

S5 Table: Statistical significance and model parameters of linear models between non-mean performance values and song diversity, as well as across song-diversity measures.

**Response**	**Predictor**	**F**	**Coef.**	**Err.**	**T**	**p**	**R^2**	**aR^2**
accuracy	Train Complexity	0.77	-0.011	0.012	-0.88	0.38	0.010	-0.0029
accuracy	Train InVar	0.46	0.014	0.020	0.68	0.49	0.0061	-0.0070
accuracy	Train SpVar	0.35	0.0090	0.015	0.59	0.55	0.0047	-0.0085
F-score	Train Complexity	2.51	-0.027	0.017	-1.58	0.11	0.032	0.019
F-score	Train InVar	3.03	0.050	0.028	1.74	0.085	0.038	0.026
F-score	Train SpVar	1.27	0.023	0.020	1.12	0.26	0.016	0.0035
precision	Train Complexity	0.37	0.0087	0.014	0.61	0.54	0.0049	-0.0082
precision	Train InVar	2.90	-0.039	0.023	-1.70	0.092	0.037	0.024
precision	Train SpVar	1.39	-0.019	0.016	-1.18	0.24	0.018	0.0052
recall	Train Complexity	5.18	-0.047	0.021	-2.27	*0.025*	0.064	0.052
recall	Train InVar	8.79	0.10	0.034	2.96	*0.0040*	0.10	0.092
recall	Train SpVar	4.28	0.052	0.025	2.07	*0.041*	0.054	0.041
S.E.R.	Train Complexity	1.10	-6.01	5.71	-1.05	0.29	0.014	0.0013
S.E.R.	Train InVar	0.26	4.91	9.57	0.51	0.60	0.0035	-0.0097
S.E.R.	Train SpVar	0.77	6.02	6.83	0.88	0.38	0.010	-0.0029
Train Complexity	Train Hypervolume (mean)	4.48	2.09	0.99	2.11	*0.037*	0.056	0.043
Train Complexity	Train Hypervolume (sd)	8.49	2.75	0.94	2.91	*0.0046*	0.10	0.089
Train Complexity	Train InVar	19.88	-0.76	0.17	-4.45	**2.84×10^{-5}**	0.20	0.19
Train Complexity	Train SpVar	238.80	-1.04	0.067	-15.45	**2.00×10^{-16}**	0.76	0.75
Train InVar	Train Hypervolume (mean)	97.84	-3.99	0.40	-9.89	**3.06×10^{-15}**	0.56	0.56
Train InVar	Train Hypervolume (sd)	138.50	-4.18	0.35	-11.76	**2.00×10^{-16}**	0.64	0.64
Train InVar	Train SpVar	40.85	0.42	0.066	6.39	**1.25×10^{-8}**	0.35	0.34
Train SpVar	Train Hypervolume (mean)	17.05	-3.18	0.77	-4.12	**9.34×10^{-5}**	0.18	0.17
Train SpVar	Train Hypervolume (sd)	16.88	-3.10	0.75	-4.10	**0.00010**	0.18	0.17
Training.Time	Train Complexity	48.94	-6436	920	-6.99	**9.46×10^{-10}**	0.39	0.38
Training.Time	Train InVar	0.44	-1317	1964	-0.67	0.505	0.0059	-0.0072
Training.Time	Train SpVar	70.32	8505	1014	8.38	**2.20×10^{-12}**	0.48	0.47

Degrees of freedom for all analyses in this table is 75. "S.E.R" = absolute model segment error rate. "Sd" = standard deviation. "InVar" = individual variation. "SpVar" = species variation. Note that Complexity, InVar, and SpVar are taken from Medina and Francis 2012 [Ref 68]. "Coef" = coefficient value. "Err" = standard error of coefficient. "aR^2" = adjusted R^2 value.

"italics" = near significant ($p < 0.05$)

"bold" = significant ($p < 0.000413$)

S6 Table: Statistical significance and model parameters of linear models between non-mean performance values and hypervolume metrics.

Response	Predictor	F	Coef.	Err.	T	p	R^2	aR^2
accuracy	Train Hypervolume (mean)	6.09	-0.11	0.046	-2.46	*0.015*	0.053	0.044
accuracy	Train Hypervolume (sd)	2.85	-0.16	0.095	-1.68	0.094	0.025	0.016
F-score	Train Hypervolume (mean)	21.24	-0.30	0.065	-4.60	**$1.11x10^{-5}$**	0.16	0.15
F-score	Train Hypervolume (sd)	10.37	-0.44	0.13	-3.22	*0.0016*	0.08764	0.079
precision	Train Hypervolume (mean)	8.76	-0.16	0.055	-2.96	*0.0037*	0.075	0.066
precision	Train Hypervolume (sd)	0.029	-0.020	0.11	-0.17	0.86	0.00027	-0.0089
recall	Train Hypervolume (mean)	19.7	-0.35	0.079	-4.43	**$2.20x10^{-5}$**	0.15	0.14
recall	Train Hypervolume (sd)	15.22	-0.64	0.16	-3.90	**0.00016**	0.12	0.11
S.E.R.	Train Hypervolume (mean)	1.47	-25.88	21.35	-1.21	0.22	0.013	0.0042
S.E.R.	Train Hypervolume (sd)	1.91	-59.86	43.22	-1.38	0.16	0.017	0.0083
Train Hypervolume (mean)	Train Hypervolume (sd)	95.89	1.39	0.14	9.79	*$2.00x10^{-16}$*	0.47	0.46
Training.Time	Train Hypervolume (mean)	33.77	24080	4144	5.81	*$6.36x10^{-8}$*	0.23	0.23
Training.Time	Train Hypervolume (sd)	3.52	17787	9478	1.87	0.063	0.031	0.022

Degrees of freedom for all analyses in this table is 108. “S.E.R” = absolute model segment error rate. “Sd” = standard deviation. “Coef” = coefficient value. “Err” = standard error of coefficient. “aR^2” = adjusted R^2 value.
“italics” = near significant ($p < 0.05$)
“bold” = significant ($p < 0.000413$)

S7 Table: Statistical significance and model parameters of linear models between performance metrics, divergence time, training time, and sample size.

Response	Predictor	F	Coef.	Err.	T	p	R^2	aR^2
accuracy	accuracy (mean)	6054	1.12	0.014	77.81	**2.00x10^{-16}**	0.98	0.98
accuracy	F-score	465.40	0.58	0.027	21.57	**2.00x10^{-16}**	0.79	0.79
accuracy	precision	21.31	0.32	0.070	4.616	**9.95x10^{-6}**	0.15	0.14
accuracy	recall	348.40	0.47	0.025	18.67	**2.00x10^{-16}**	0.74	0.74
accuracy	Testing Test Size	0.054	-0.0052	0.022	-0.23	0.816	0.00045	-0.0079
accuracy	Testing Train Size	6.36	-0.063	0.025	-2.52	*0.013*	0.050	0.042
accuracy	Testing Val Size	10.51	-0.10	0.031	-3.24	*0.0015*	0.081	0.073
accuracy	Training Test Size	6.74	0.057	0.022	2.59	*0.010*	0.053	0.045
accuracy	Training Train Size	0.049	0.0057	0.025	0.22	0.82	0.00041	-0.0079
accuracy	Training Val Size	0.21	0.015	0.033	0.46	0.64	0.0017	-0.0066
accuracy	Uncorrected accuracy	1698	1.22	0.029	41.20	**2.00x10^{-16}**	0.93	0.93
F-score	F-score (mean)	2806	0.99	0.018	52.97	**2.00x10^{-16}**	0.95	0.95
F-score	precision	49.04	0.68	0.097	7.00	**1.61x10^{-10}**	0.29	0.28
F-score	recall	742.80	0.77	0.028	27.25	**2.00x10^{-16}**	0.86	0.86
F-score	YEAR	71.68	-0.0047	0.00056	-8.46	**7.71x10^{-14}**	0.37	0.37
precision	precision (mean)	246.60	0.89	0.057	15.7	**2.00x10^{-16}**	0.67	0.67
precision	recall	6.54	0.15	0.058	2.55	*0.011*	0.052	0.044
precision	YEAR	35.65	-0.0029	0.00049	-5.97	**2.51x10^{-8}**	0.23	0.22
recall	recall (mean)	3681	1.03	0.017	60.66	**2.00x10^{-16}**	0.96	0.96
recall	YEAR	44.26	-0.0048	0.00073	-6.65	**9.25x10^{-10}**	0.27	0.26
S.E.R.	accuracy	0.014	4.92	40.76	0.12	0.90	0.00012	-0.0082
S.E.R.	F-score	0.070	-7.15	26.88	-0.26	0.79	0.00059	-0.0078
S.E.R.	precision	33.88	-175.37	30.13	-5.82	**5.07x10^{-8}**	0.22	0.21
S.E.R.	recall	3.51	41.61	22.18	1.87	0.063	0.028	0.020
S.E.R.	S.E.R. (mean)	149.5	0.47	0.038	12.22	**2.00x10^{-16}**	0.55	0.55
S.E.R.	YEAR	10.24	0.64	0.20	3.20	*0.0017*	0.079	0.071
S.E.R. (Neg)	YEAR	4.03	0.45	0.22	2.00	*0.046*	0.032	0.024
Training Time	Training Train Size	42.54	0.13	2.09	6.52	**1.76x10^{-9}**	0.26	0.25
Training Time	Training Train Size (log)	72.79	0.00038	4475	8.53	**5.43x10^{-14}**	0.37	0.37
Uncorrected accuracy	YEAR	53.35	-0.0022	0.00030	-7.30	**3.45x10^{-11}**	0.30	0.30
Uncorrected S.E.R.	YEAR	12.87	0.10	0.027	3.58	*0.00048*	0.097	0.090

Degrees of freedom for all analyses in this table is 119. "S.E.R" = absolute model segment error rate. "S.E.R (Neg)" = model segment error rate including negative values as negative. "Sd" = standard deviation. "Coef" = coefficient value. "Err" = standard error of coefficient. "aR^2" = adjusted R^2 value. Uncorrected values are taken before removing predicted syllables that are too short.

"italics" = near significant ($p < 0.05$)

"bold" = significant ($p < 0.000413$)

S8 Table: Species-specific correlations between divergence time and performance of machine learning models.

Response	Predictor	Species	F	Coef.	Err.	T	p	R^2	aR^2
accuracy	YEAR	9SPP	12.99	-0.0027	0.00075	-3.60	*0.0057*	0.59	0.54
S.E.R.	YEAR	9SPP	0.74	-0.16	0.18	-0.86	0.41	0.076	-0.026
accuracy	YEAR	CA	5.56	-0.0046	0.0019	-2.35	*0.042*	0.38	0.31
S.E.R.	YEAR	CA	0.00077	-0.020	0.719	-0.028	0.98	$8.62x10^{-5}$	-0.11
accuracy	YEAR	CC	3.58	-0.0020	0.0010	-1.89	0.091	0.28	0.21
S.E.R.	YEAR	CC	0.25	-0.084	0.16	-0.49	0.63	0.026	-0.081
accuracy	YEAR	CS	0.47	-0.0022	0.00078	-2.84	0.19	0.47	0.41
S.E.R.	YEAR	CS	0.51	0.071	0.10	0.71	0.49	0.053	-0.051
accuracy	YEAR	EV	1.28	-0.00074	0.00065	-1.13	0.28	0.12	0.027
S.E.R.	YEAR	EV	1.16	0.63	0.59	1.08	0.30	0.11	0.016
accuracy	YEAR	MF	16.91	-0.0014	0.00035	-4.11	*0.0026*	0.65	0.61
S.E.R.	YEAR	MF	0.35	0.097	0.16	0.59	0.56	0.038	-0.068
accuracy	YEAR	MT	3.55	-0.0029	0.0015	-1.88	0.092	0.28	0.20
S.E.R.	YEAR	MT	3.51	1.85	0.98	1.87	0.093	0.28	0.20
accuracy	YEAR	PA	9.14	-0.0022	0.00073	-3.02	*0.014*	0.50	0.44
S.E.R.	YEAR	PA	1.55	0.20	0.16	1.24	0.24	0.14	0.052
accuracy	YEAR	PC	12.75	-0.0024	0.00069	-3.57	*0.0060*	0.58	0.54
S.E.R.	YEAR	PC	1.35	0.79	0.68	1.16	0.27	0.13	0.034
accuracy	YEAR	VA	4.01	-0.0012	0.00062	-2.004	*0.076*	0.30	0.23
S.E.R.	YEAR	VA	0.090	0.27	0.90	0.30	0.77	0.0099	-0.10
accuracy	YEAR	ZL	17.05	-0.0027	0.00067	-4.12	*0.0025*	0.65	0.61
S.E.R.	YEAR	ZL	2.52	-0.18	0.11	-1.58	0.14	0.21	0.13

Degrees of freedom for all analyses in this table is 9. “S.E.R” = absolute model segment error rate. “Coef” = coefficient value. “Err” = standard error of coefficient. “aR^2” = adjusted R^2 value.
“italics” = near significant ($p < 0.05$)
“bold” = significant ($p < 0.000413$)

S9 Table: Individual songs used in models.

Collection	ID	Genus	Species	Latitude	Longitude	Year	Train (s)	Val (s)	Test (s)
BLB	28330	*Calypte*	*anna*	39.75	-122.6667	1995.00	4.45483	0	4.1875
BLB	33172	*Calypte*	*anna*	34.7667	-118.3833	1980.00	6.64037	0	3.4773
BLB	33565	*Calypte*	*anna*	37.7289	-122.4948	1990.00	6.79648	0	0
BLB	34484	*Calypte*	*anna*	37.7736	-122.4622	1994.00	4.25785	4.2277	0
BLB	34878	*Calypte*	*anna*	37.7167	-122.5	1987.00	25.60405	0	0
BLB	36231	*Calypte*	*anna*	36.5192	-121.9456	1975.00	0	13.09448	0
XC	42442	*Calypte*	*anna*	47.7839	-122.2739	2010.00	80.82277	19.18344	15.17483
XC	43423	*Calypte*	*anna*	47.6976	-122.2087	2010.00	39.50789	4.67124	8.36763
XC	70079	*Calypte*	*anna*	32.2784	-110.9509	2011.00	69.19425	0	0
XC	80361	*Calypte*	*anna*	34.57	-112.3753	2000.00	54.42812	15.23915	15.14803
XC	132248	*Calypte*	*anna*	31.906	-109.1543	2008.00	21.12376	0	0
XC	132250	*Calypte*	*anna*	32.7231	-116.9473	2001.00	12.34609	0	0
XC	159355	*Calypte*	*anna*	48.429528	-123.479208	2012.00	6.88827	0	0

XC	169597	*Calypte*	*anna*	34.2148	-118.1477	2014.00	24.37393	0	16.03645
XC	196689	*Calypte*	*anna*	31.912	-109.1426	2014.00	5.61125	0	0
XC	367257	*Calypte*	*anna*	39.43	-122.1862	2017.00	8.98135	0	0
XC	408456	*Calypte*	*anna*	34.201	-118.019	2015.00	55.99927	0	2.10983
XC	442358	*Calypte*	*anna*	34.8116	-115.5608	2018.00	27.09547	0	5.58043
XC	453109	*Calypte*	*anna*	32.6287	-116.923	2018.00	20.12948	13.36248	0
XC	473411	*Calypte*	*anna*	47.0562	-122.9226	2019.00	8.47885	0	0
XC	501895	*Calypte*	*anna*	31.403	-110.2577	2019.00	44.32586	0	0
XC	549602	*Calypte*	*anna*	47.7022	-122.3088	2020.00	10.33542	0	0
XC	624426	*Calypte*	*anna*	45.5575	-122.6912	2020.00	0	0	0
BLB	8	*Cardinalis*	*cardinalis*	39.9833	-82.9167	1948.00	15.57147	0	0
BLB	15	*Cardinalis*	*cardinalis*	39.9961	-83.0189	1948.00	10.14246	0	0
BLB	27	*Cardinalis*	*cardinalis*	39.9833	-82.9167	1948.00	4.51245	0	0
BLB	31	*Cardinalis*	*cardinalis*	39.9833	-82.9167	1948.00	0	6.29532	0
BLB	96	*Cardinalis*	*cardinalis*	39.9833	-82.9167	1949.00	29.23813	4.19085	4.80725
BLB	126	*Cardinalis*	*cardinalis*	39.9833	-82.9167	1949.00	23.89488	0	4.85281
BLB	295	*Cardinalis*	*cardinalis*	39.9833	-82.9167	1952.00	22.82154	4.70273	2.21368
BLB	330	*Cardinalis*	*cardinalis*	40.4	-83.05	1953.00	27.17319	4.59218	0
BLB	331	*Cardinalis*	*cardinalis*	40.1	-83	1953.00	2.89306	5.05247	0
BLB	337	*Cardinalis*	*cardinalis*	40.65	-83	1953.00	42.50011	10.39304	0
BLB	415	*Cardinalis*	*cardinalis*	39.9961	-83.0189	1953.00	9.6011	0	10.14715
BLB	547	*Cardinalis*	*cardinalis*	40.0439	-83.0261	1953.00	62.15121	5.32717	0
BLB	776	*Cardinalis*	*cardinalis*	39.9833	-82.9167	1954.00	10.32537	0	0
BLB	1098	*Cardinalis*	*cardinalis*	30.35	-82.7	1954.00	5.33856	0	0
BLB	1310	*Cardinalis*	*cardinalis*	39.35	-82.25	1955.00	4.70809	0	4.16137
BLB	1352	*Cardinalis*	*cardinalis*	39.9403	-83.0325	1955.00	15.61234	0	0
BLB	1810	*Cardinalis*	*cardinalis*	39.6667	-82.6667	1956.00	13.59028	0	0
BLB	2328	*Cardinalis*	*cardinalis*	28.1667	-81.8333	1957.00	85.63739	9.21116	16.0733
BLB	2435	*Cardinalis*	*cardinalis*	39.9403	-83.0325	1957.00	6.42865	5.69366	0
BLB	3191	*Cardinalis*	*cardinalis*	39.9403	-83.0325	1958.00	0	5.49132	5.54157
BLB	3860	*Cardinalis*	*cardinalis*	39.9833	-82.9167	1959.00	86.7047	5.44308	4.69067
BLB	3886	*Cardinalis*	*cardinalis*	39.9403	-83.0325	1959.00	3.52487	4.39319	4.04412
BLB	4405	*Cardinalis*	*cardinalis*	28.0203	-97.0642	1960.00	77.19204	3.39154	6.91574
BLB	8201	*Cardinalis*	*cardinalis*	39.9	-83.2167	1966.00	9.74783	0	0
BLB	8208	*Cardinalis*	*cardinalis*	39.9	-83.2167	1966.00	7.21925	9.41015	0
BLB	9680	*Cardinalis*	*cardinalis*	32.2692	-110.8758	1968.00	14.14839	0	0
BLB	9700	*Cardinalis*	*cardinalis*	32.2692	-110.8758	1968.00	7.571	0	0
BLB	11276	*Cardinalis*	*cardinalis*	40.0833	-82.9167	1971.00	10.75484	0	0
BLB	11634	*Cardinalis*	*cardinalis*	40.0833	-82.9167	1972.00	5.31846	4.87693	5.31377

BLB	11876	*Cardinalis*	*cardinalis*	35.3333	-98.1833	1972.00	5.50874	9.75319	0
BLB	13730	*Cardinalis*	*cardinalis*	39.9	-83.2167	1976.00	5.49668	0	0
BLB	14392	*Cardinalis*	*cardinalis*	39.9961	-83.0189	1977.00	7.18441	0	0
BLB	14393	*Cardinalis*	*cardinalis*	39.9961	-83.0189	1977.00	0	3.8927	0
BLB	14394	*Cardinalis*	*cardinalis*	39.9961	-83.0189	1977.00	8.40314	0	0
BLB	15027	*Cardinalis*	*cardinalis*	39.9	-83.2167	1979.00	11.98563	0	6.69531
BLB	15723	*Cardinalis*	*cardinalis*	37.9167	-75.5	1981.00	8.59275	0	0
BLB	15779	*Cardinalis*	*cardinalis*	39.9	-83.2167	1982.00	12.34877	0	0
BLB	16180	*Cardinalis*	*cardinalis*	40.0833	-82.9167	1984.00	4.6565	0	0
BLB	16188	*Cardinalis*	*cardinalis*	40.0833	-82.9167	1984.00	8.01588	0	0
BLB	16287	*Cardinalis*	*cardinalis*	40.0833	-82.9167	1985.00	9.50864	0	0
BLB	16292	*Cardinalis*	*cardinalis*	39.9	-83.2167	1985.00	14.46597	2.27264	0
BLB	16303	*Cardinalis*	*cardinalis*	39.9	-83.2167	1985.00	14.98991	0	0
BLB	16305	*Cardinalis*	*cardinalis*	39.9	-83.2167	1985.00	7.80282	0	0
BLB	21917	*Cardinalis*	*cardinalis*	39.9961	-83.0189	1966.00	8.17467	0	0
BLB	21956	*Cardinalis*	*cardinalis*	39.9403	-83.0325	1966.00	13.48442	0	0
BLB	21960	*Cardinalis*	*cardinalis*	39.9403	-83.0325	1966.00	6.04072	0	0
BLB	21982	*Cardinalis*	*cardinalis*	39.9961	-83.0189	1966.00	3.96841	3.78952	0
BLB	21993	*Cardinalis*	*cardinalis*	39.9961	-83.0189	1966.00	10.7535	0	0
BLB	21997	*Cardinalis*	*cardinalis*	39.9961	-83.0189	1966.00	5.91476	0	0
BLB	22008	*Cardinalis*	*cardinalis*	39.9961	-83.0189	1966.00	3.82168	0	0
BLB	22016	*Cardinalis*	*cardinalis*	39.9961	-83.0189	1967.00	0	0	0
BLB	22026	*Cardinalis*	*cardinalis*	39.9961	-83.0189	1967.00	0	0	5.2327
BLB	22036	*Cardinalis*	*cardinalis*	39.9961	-83.0189	1967.00	1.5678	0	9.89523
BLB	22049	*Cardinalis*	*cardinalis*	39.9961	-83.0189	1967.00	8.72273	0	0
BLB	22055	*Cardinalis*	*cardinalis*	39.9961	-83.0189	1967.00	11.09654	0	0
BLB	22085	*Cardinalis*	*cardinalis*	39.9961	-83.0189	1967.00	3.83575	0	4.43272
BLB	22096	*Cardinalis*	*cardinalis*	39.9961	-83.0189	1967.00	10.55786	0	0
BLB	22118	*Cardinalis*	*cardinalis*	39.2961	-82.6567	1967.00	0	0	0
BLB	22125	*Cardinalis*	*cardinalis*	39.9961	-83.0189	1967.00	6.77303	0	0
BLB	22137	*Cardinalis*	*cardinalis*	39.9961	-83.0189	1968.00	5.30305	0	0
BLB	22581	*Cardinalis*	*cardinalis*	53.8333	-79.1667	1957.00	11.0148	0	0
BLB	32203	*Cardinalis*	*cardinalis**	31.8833	-109.2	2001.00	17.13994	0	0
BLB	32204	*Cardinalis*	*cardinalis**	31.8833	-109.2	2001.00	19.27523	0	0
BLB	5539	*Cardinalis*	*sinuatus*	28.1	-97.4833	1962.00	18.11479	5.00892	0
BLB	5541	*Cardinalis*	*sinuatus*	28.1	-97.4833	1962.00	59.98376	7.27419	8.27182
BLB	5584	*Cardinalis*	*sinuatus*	28.1	-97.4833	1962.00	47.70802	7.54085	17.84947
BLB	9728	*Cardinalis*	*sinuatus*	31.8522	-110.9742	1968.00	36.75352	0	0
BLB	9739	*Cardinalis*	*sinuatus*	32.3561	-111.0878	1968.00	11.47308	0	5.86987

BLB	10213	*Cardinalis*	*sinuatus*	32.3333	-110.7	1969.00	25.47742	6.61424	0
BLB	10721	*Cardinalis*	*sinuatus*	31.6675	-100.7167	1970.00	23.92704	4.79921	0
BLB	24840	*Cardinalis*	*sinuatus*	31.6333	-111.0333	1997.00	96.26694	8.78906	8.7569
BLB	196	*Empidonax*	*virescens*	39.9833	-82.9167	1950.00	9.39072	4.53523	0
BLB	5160	*Empidonax*	*virescens*	40.0833	-82.9167	1961.00	16.95636	2.12323	1.35474
BLB	6405	*Empidonax*	*virescens*	40.0167	-83.1	1963.00	4.87291	2.42875	0
BLB	10661	*Empidonax*	*virescens*	39.5797	-82.5222	1970.00	6.38577	0	0
BLB	11793	*Empidonax*	*virescens*	39.3333	-84.25	1972.00	4.62434	0	2.30413
BLB	13771	*Empidonax*	*virescens*	39.9	-83.2167	1976.00	9.1522	2.2177	2.20966
BLB	14455	*Empidonax*	*virescens*	40.0833	-82.9167	1977.00	4.80591	0	0
BLB	15125	*Empidonax*	*virescens*	40.4	-83.05	1979.00	0	0	2.35103
BLB	16639	*Empidonax*	*virescens*	39.5833	-83.9833	1987.00	4.74762	5.19451	2.37247
XC	33587	*Empidonax*	*virescens*	36.0403	-93.3406	2009.00	31.8317	0	7.61455
XC	33588	*Empidonax*	*virescens*	36.0403	-93.3406	2009.00	24.32904	2.41066	0
XC	44373	*Empidonax*	*virescens*	42.91164591	-88.47706532	2009.00	1.72659	0	0
XC	182091	*Empidonax*	*virescens*	42.6575	-80.473	2001.00	2.32624	0	2.32825
XC	286544	*Empidonax*	*virescens*	29.8235	-99.5773	2015.00	18.87524	0	0
XC	316116	*Empidonax*	*virescens*	34.112	-91.0917	2016.00	6.61089	0	0
XC	324931	*Empidonax*	*virescens*	42.1262	-79.5069	2016.00	6.98073	4.6498	2.37984
XC	370169	*Empidonax*	*virescens*	32.3861	-85.3675	2017.00	32.52448	3.10478	4.15668
XC	371473	*Empidonax*	*virescens*	38.802	-94.6896	2017.00	4.75834	0	1.99459
XC	371474	*Empidonax*	*virescens*	38.802	-94.6896	2017.00	9.12875	0	0
XC	372010	*Empidonax*	*virescens*	38.802	-94.6896	2017.00	16.5222	2.37046	0
XC	420873	*Empidonax*	*virescens*	42.3097	-76.5008	2018.00	13.97553	0	0
XC	501241	*Empidonax*	*virescens*	38.3619	-77.3422	2017.00	2.25991	0	0
BLB	7184	*Melozone*	*fusca*	32.0167	-109.1667	1964.00	6.20822	3.32387	19.66115
BLB	10118	*Melozone*	*fusca*	32.6333	-110.95	1969.00	5.99583	4.17477	25.25163
BLB	10228	*Melozone*	*fusca*	31.5394	-110.7556	1969.00	7.16297	0	21.39243
BLB	17134	*Melozone*	*fusca*	31.85	-110.9	1989.00	8.43932	1.8626	22.66945
BLB	22880	*Melozone*	*fusca*	32.2167	-110.9167	?	7.36531	0	14.19998
BLB	40252	*Melozone*	*fusca*	34.1622	-118.1931	1974.00	0	0	3.99856
XC	18127	*Melozone*	*fusca*	32.43619	-110.90482	2008.00	3.29305	0	16.32589
XC	217984	*Melozone*	*fusca*	31.7414	-110.8873	2012.00	15.54936	0	22.23998
XC	361866	*Melozone*	*fusca*	38.2545	-104.7367	2015.00	3.34196	0	10.67511
XC	530767	*Melozone*	*fusca*	19.2553	-99.0259	2020.00	0	0	3.71917
XC	566741	*Melozone*	*fusca*	32.3267	-110.7033	2020.00	7.34119	0	6.94053
XC	579549	*Melozone*	*fusca*	19.1637	-99.1696	2020.00	6.58141	0	20.31641
XC	1663	*Myiarchus*	*tuberculifer*	-22.0403	-64.5584	1992	8.46143	0	0
XC	1721	*Myiarchus*	*tuberculifer*	-22.1001	-64.4334	1992	6.03335	0	0

XC	76805	*Myiarchus*	*tuberculifer*	14.5859	-90.4678	2010	5.33856	0	0
XC	125735	*Myiarchus*	*tuberculifer*	14.6724	-91.485	2013	8.10365	0	0
XC	331718	*Myiarchus*	*tuberculifer*	18.0357	-94.4722	2016	3.68232	0	0
XC	373645	*Myiarchus*	*tuberculifer*	13.9475	-87.1811	2017	14.49076	0	2.58687
XC	429409	*Myiarchus*	*tuberculifer*	21.4302	-87.3345	2018	9.51936	6.73752	0
XC	22439	*Myiarchus*	*tuberculifer*	0.1431	-79.1334	2008	5.1054	0	3.9798
XC	465235	*Myiarchus*	*tuberculifer*	-3.649	-79.749	2017	8.24569	0	2.52925
XC	441631	*Myiarchus*	*tuberculifer*	28.3714	-109.0318	2018	33.13217	2.5326	0
XC	352213	*Myiarchus*	*tuberculifer*	23.589	-105.8694	2015	22.99373	7.69897	2.73695
XC	125996	*Myiarchus*	*tuberculifer*	?	?	2011	12.10154	0	0
BLB	32001	*Myiarchus*	*tuberculifer*	32.2072	-109.3681	1981.00	2.34165	0	2.03077
XC	11573	*Myiarchus*	*tuberculifer*	29.287	-108.1501	2006	0	0	4.19018
XC	15530	*Myiarchus*	*tuberculifer*	10.67	-85.5	1993	10.57327	0	0
XC	38922	*Myiarchus*	*tuberculifer*	-5.671292	-77.758827	2006	2.05355	4.49302	0
XC	41477	*Myiarchus*	*tuberculifer*	-6.3014	-79.4623	2001	6.41592	0	0
XC	41478	*Myiarchus*	*tuberculifer*	-6.3014	-79.4623	2001	0	0	5.94893
XC	82051	*Myiarchus*	*tuberculifer*	4.6834	-74.3834	2009	3.66155	0	0
XC	219903	*Myiarchus*	*tuberculifer*	8.299	-70.0851	2011	5.25816	1.60532	0
XC	268876	*Myiarchus*	*tuberculifer*	13.9641	-87.1881	2015	8.00382	0	2.64181
XC	279000	*Myiarchus*	*tuberculifer*	31.5403	-110.3754	2015	1.30248	0	0
XC	299054	*Myiarchus*	*tuberculifer*	-15.8816	-38.9001	2012	5.10741	2.66727	5.03572
XC	335886	*Myiarchus*	*tuberculifer*	12.9006	-86.5645	2014	7.16967	0	0
XC	360563	*Myiarchus*	*tuberculifer*	19.516	-96.938	2016	3.10143	0	0
XC	382467	*Myiarchus*	*tuberculifer*	9.9261	-75.1074	2017	2.16075	0	0
XC	432855	*Myiarchus*	*tuberculifer*	-9.5975	-55.9325	2018	8.30264	0	0
XC	435556	*Myiarchus*	*tuberculifer*	6.5574	-75.8593	2017	2.9212	0	0
XC	504356	*Myiarchus*	*tuberculifer*	-19.1456	-40.0645	2019	9.09927	0	0
XC	560657	*Myiarchus*	*tuberculifer*	31.9089	-109.2517	2017	13.1789	0	0
XC	596145	*Myiarchus*	*tuberculifer*	-3.5761	-79.5781	2019	7.83431	0	0
XC	672687	*Myiarchus*	*tuberculifer*	19.4577	-103.6175	2021.00	4.18884	0	0
XC	708791	*Myiarchus*	*tuberculifer*	-9.4642	-50.0895	1999.00	5.41561	5.42566	0
XC	727101	*Myiarchus*	*tuberculifer*	25.766	-100.2271	2022.00	6.35093	0	0
BLB	7710	*Passerina*	*amoena*	44.6125	-123.2403	1965.00	24.85432	4.20224	8.66913
BLB	9155	*Passerina*	*amoena*	47.325	-114.225	1967.00	8.56059	0	0
BLB	18499	*Passerina*	*amoena*	39.6333	-120.5167	1992.00	4.03273	0	3.98985
BLB	38416	*Passerina*	*amoena*	41.7412	-111.7928	1974.00	2.47833	0	3.28032
XC	1277	*Passerina*	*amoena*	38.668892	-108.304596	1991.00	3.17044	0	0
XC	36565	*Passerina*	*amoena*	48.4806	-121.5834	2009.00	7.98707	0	0
XC	36566	*Passerina*	*amoena*	48.4806	-121.5834	2009.00	10.12839	0	0

XC	62201	*Passerina*	*amoena*	40.137	-121.448	2010.00	9.2929	0	0
XC	107702	*Passerina*	*amoena*	47.014	-120.706	2012.00	9.23394	0	0
XC	137571	*Passerina*	*amoena*	49.0346	-119.5677	2013.00	2.45019	0	0
XC	137623	*Passerina*	*amoena*	49.2997	-119.5327	2013.00	3.51147	0	0
XC	154997	*Passerina*	*amoena*	49.4905	-119.6205	2010.00	9.2661	0	0
XC	185599	*Passerina*	*amoena*	39.9325	-105.2769	2014.00	0	4.5292	0
XC	251809	*Passerina*	*amoena*	49.0804	-113.8905	2015.00	6.71541	0	0
XC	252848	*Passerina*	*amoena*	31.7572	-110.8438	2015.00	8.94249	0	4.08901
XC	255825	*Passerina*	*amoena*	49.0459	-113.7889	2015.00	3.94965	0	0
XC	269196	*Passerina*	*amoena*	46.8437	-120.7069	2015.00	4.06355	0	0
XC	269198	*Passerina*	*amoena*	46.8437	-120.7069	2015.00	4.29269	0	0
XC	326482	*Passerina*	*amoena*	38.7485	-121.6803	2016.00	0	0	3.45184
XC	331695	*Passerina*	*amoena*	40.2286	-105.2903	2016.00	3.12555	0	0
XC	333586	*Passerina*	*amoena*	38.4422	-119.0056	2016.00	0	4.14328	0
XC	333587	*Passerina*	*amoena*	38.4422	-119.0056	2016.00	6.39113	0	0
XC	376094	*Passerina*	*amoena*	46.8897	-120.7962	2017.00	3.66825	7.23734	0
XC	386101	*Passerina*	*amoena*	45.0765	-108.5295	2017.00	3.25754	0	1.90146
XC	386105	*Passerina*	*amoena*	45.0765	-108.5295	2017.00	3.4103	0	0
XC	415452	*Passerina*	*amoena*	44.3896	-123.3033	2018.00	2.75705	0	2.80462
XC	419780	*Passerina*	*amoena*	48.9207	-122.2617	2018.00	4.90775	0	0
XC	421763	*Passerina*	*amoena*	46.6453	-122.9843	2018.00	7.42628	0	0
XC	468976	*Passerina*	*amoena*	38.1378	-122.6037	2019	3.0351	0	0
XC	478598	*Passerina*	*amoena*	48.3113	-121.5486	2019	7.78004	4.36304	0
XC	478599	*Passerina*	*amoena*	48.3114	-121.5486	2019	6.92646	0	0
XC	483706	*Passerina*	*amoena*	38.7985	-120.3483	2019	7.59378	3.45921	0
XC	548225	*Passerina*	*amoena*	33.3543	-114.6935	2020	3.44648	0	0
XC	559822	*Passerina*	*amoena*	32.6675	-116.9268	2020	12.62749	0	0
XC	563913	*Passerina*	*amoena*	40.2286	-105.2903	2020	3.81632	0	0
XC	582683	*Passerina*	*amoena*	38.5431	-121.6201	2020	6.68593	0	0
XC	642626	*Passerina*	*amoena*	48.4991	-114.1331	2017.00	6.13385	0	0
XC	647816	*Passerina*	*amoena*	48.4812	-123.3732	2021.00	6.41726	0	0
XC	651826	*Passerina*	*amoena*	38.7563	-122.7701	2021.00	7.63331	3.29372	0
XC	662233	*Passerina*	*amoena*	44.7361	-123.1474	2021.00	6.32614	0	0
XC	662824	*Passerina*	*amoena*	40.001	-119.8642	2019.00	6.85812	0	0
XC	665966	*Passerina*	*amoena*	40.1864	-105.3361	2021.00	6.33686	0	0
XC	716430	*Passerina*	*amoena*	34.2139	-118.3069	2022.00	0	0	3.53425
XC	722862	*Passerina*	*amoena*	39.3136	-122.9465	2022.00	7.38742	0	0
XC	56591	*Poecile*	*carolinensis*	29.649	-95.014	2010.00	23.70259	0	2.5996
XC	57321	*Poecile*	*carolinensis*	29.649	-95.014	2010.00	10.48215	3.06123	0

XC	42453	*Poecile*	*carolinensis*	34.1712	-84.2017	2009.00	6.76097	0	0
XC	734763	*Poecile*	*carolinensis*	28.0735	-82.3744	2022.00	25.48211	3.36675	8.09762
BLB	44185	*Poecile*	*carolinensis*	37.6418	-89.1468	2014.00	6.50101	0	0
BLB	44186	*Poecile*	*carolinensis*	35.9786	-84.9637	2014.00	3.42906	0	0
BLB	175	*Poecile*	*carolinensis*	40.1243	-83.0258	1950.00	0	3.3969	0
BLB	179	*Poecile*	*carolinensis*	40.1243	-83.0258	1950.00	6.16735	3.29975	0
BLB	1312	*Poecile*	*carolinensis*	40.0833	-82.9167	1955.00	3.35737	0	2.28537
BLB	5112	*Poecile*	*carolinensis*	39.6667	-82.6667	1961.00	3.35402	0	3.39958
BLB	11767	*Poecile*	*carolinensis*	39.9	-83.2167	1972.00	3.40293	0	0
BLB	11911	*Poecile*	*carolinensis*	39.9	-83.2167	1973.00	3.33995	2.59491	3.32655
BLB	13003	*Poecile*	*carolinensis*	39.5797	-82.5222	1974.00	0	3.17647	0
BLB	15448	*Poecile*	*carolinensis*	39.9	-83.2167	1980.00	5.16972	0	0
BLB	16251	*Poecile*	*carolinensis*	39.2667	-82.4	1984.00	9.82622	2.28135	0
BLB	16830	*Poecile*	*carolinensis*	40.55	-82.1333	1988.00	13.25662	0	0
BLB	27766	*Poecile*	*carolinensis*	39.5797	-82.5222	2002.00	2.13663	3.0083	0
BLB	40572	*Poecile*	*carolinensis*	40.1389	-83.3128	1989.00	0	3.32387	0
XC	15183	*Poecile*	*carolinensis*	35.2176	-85.922	2007.00	6.63501	0	0
XC	70980	*Poecile*	*carolinensis*	29.5826	-99.7309	2010.00	8.174	0	3.06659
XC	254933	*Poecile*	*carolinensis*	32.032	-97.1212	2015.00	4.95331	2.94666	2.95738
XC	267591	*Poecile*	*carolinensis*	32.2517	-97.8147	2015.00	8.33547	0	0
XC	309925	*Poecile*	*carolinensis*	34.3665	-89.5192	2016.00	9.26275	0	3.30377
XC	310196	*Poecile*	*carolinensis*	38.0915	-78.4939	2016.00	0	0	3.36675
XC	385887	*Poecile*	*carolinensis*	39.9826	-74.2374	2017	1.26831	0	0
XC	416023	*Poecile*	*carolinensis*	38.7877	-77.2332	2018.00	6.40922	0	3.17781
XC	417997	*Poecile*	*carolinensis*	40.5652	-83.6255	2018.00	9.96692	0	0
XC	434624	*Poecile*	*carolinensis*	34.9961	-82.4037	2018.00	14.85658	0	0
XC	458045	*Poecile*	*carolinensis*	37.2602	-97.4172	2019.00	12.8506	0	0
XC	483022	*Poecile*	*carolinensis*	34.0091	-86.1929	2019	3.54296	0	1.85992
XC	499562	*Poecile*	*carolinensis*	38.3619	-77.3422	2017	11.94744	0	3.01232
XC	616885	*Poecile*	*carolinensis*	37.9754	-90.8983	2021.00	5.71443	0	0
XC	438079	*Vireo*	*altiloquus*	18.4485	-69.0962	2018.00	10.06072	2.5661	5.58713
XC	489358	*Vireo*	*altiloquus*	25.2792	-80.2983	2019.00	16.482	5.20255	0
XC	495838	*Vireo*	*altiloquus*	13.211	-59.5969	2019.00	35.63596	0	1.5812
XC	82985	*Vireo*	*altiloquus*	22.6689	-83.4789	2011.00	2.54332	3.74932	0
XC	82986	*Vireo*	*altiloquus*	22.6689	-83.4789	2011.00	23.52906	0	5.53889
XC	82987	*Vireo*	*altiloquus*	21.01	-77.7198	2011.00	15.47298	2.50178	2.54332
XC	104633	*Vireo*	*altiloquus*	24.6959	-81.3209	2012.00	39.58025	7.33047	5.04845
XC	104634	*Vireo*	*altiloquus*	24.6959	-81.3209	2012.00	39.8114	2.58754	5.4337
XC	256744	*Vireo*	*altiloquus*	22.657	-83.445	2015.00	23.99806	2.86961	2.90378

XC	97198	*Vireo*	*altiloquus*	18.492755	-69.952826	2012.00	16.25353	2.61769	2.55002
XC	215245	*Vireo*	*altiloquus*	17.6285	-63.2493	2013.00	4.73422	0	1.67969
XC	370027	*Vireo*	*altiloquus*	18.0442	-66.114	2017.00	2.85152	0	0
BLB	13760	*Zonotrichia*	*leucophrys*	40.0833	-82.9167	1976.00	11.39603	3.72587	16.78618
BLB	18611	*Zonotrichia*	*leucophrys*	61.75	-150.0833	1993.00	55.73261	7.37134	7.05979
BLB	18613	*Zonotrichia*	*leucophrys*	61.5294	-149.9075	1993.00	29.48201	12.71593	3.72453
BLB	18799	*Zonotrichia*	*leucophrys*	61.25	-149.25	1993.00	43.81264	3.75937	14.9678
BLB	23095	*Zonotrichia*	*leucophrys*	51.1667	-80.3333	1958.00	17.50576	4.2076	0
BLB	23096	*Zonotrichia*	*leucophrys*	51.1667	-80.3333	1958.00	23.2691	7.7385	0
BLB	23097	*Zonotrichia*	*leucophrys*	58.725	-94.1167	1959.00	20.67285	0	0
BLB	23875	*Zonotrichia*	*leucophrys*	38.5	-121.75	1997.00	34.72744	4.16539	12.55111
BLB	23876	*Zonotrichia*	*leucophrys*	38.5	-121.75	1997.00	39.39265	4.38984	4.08968
BLB	23877	*Zonotrichia*	*leucophrys*	38.5	-121.75	1997.00	20.84839	0	4.20358
BLB	23903	*Zonotrichia*	*leucophrys*	58.7333	-93.8167	1997.00	12.08814	4.11112	4.11112
BLB	24704	*Zonotrichia*	*leucophrys*	38.0833	-122.9167	1998.00	80.84354	12.65965	39.46367
BLB	29924	*Zonotrichia*	*leucophrys*	38.5	-121.75	1990.00	12.95646	4.221	0
BLB	33624	*Zonotrichia*	*leucophrys*	33.2167	-116.25	1976.00	30.53056	0	4.01196
BLB	35099	*Zonotrichia*	*leucophrys*	60.5711	-151.2478	1968.00	7.82359	0	10.29321
BLB	35101	*Zonotrichia*	*leucophrys*	61.5833	-149.1	1968.00	11.27878	3.50946	0
BLB	35102	*Zonotrichia*	*leucophrys*	61.0561	-149.7972	1968.00	11.10056	3.46524	0
BLB	35130	*Zonotrichia*	*leucophrys*	34.4125	-119.8481	1969.00	6.36835	0	0
BLB	35608	*Zonotrichia*	*leucophrys*	37.8775	-122.2508	1969.00	6.27254	0	7.102
BLB	37698	*Zonotrichia*	*leucophrys*	49.0878	-120.8166	1970.00	21.85808	0	0
BLB	37953	*Zonotrichia*	*leucophrys*	37.2502	-119.7513	1987.00	14.6998	0	5.49266
BLB	41400	*Zonotrichia*	*leucophrys*	63.2941	-148.5528	1996.00	12.18797	0	8.19678
BLB	42491	*Zonotrichia*	*leucophrys*	34.4423	-119.8112	1967.00	6.28527	0	0
BLB	42554	*Zonotrichia*	*leucophrys*	64.8548	-147.8379	1973.00	8.44736	0	8.42994
BLB	2529	*Zonotrichia*	*leucophrys*	39.6667	-82.6667	1957.00	57.72117	0	4.44746
BLB	2570	*Zonotrichia*	*leucophrys*	39.6667	-82.6667	1957.00	51.33607	3.90476	3.57311
BLB	5132	*Zonotrichia*	*leucophrys*	39.9403	-83.0325	1961.00	63.27949	0	4.14998
BLB	5769	*Zonotrichia*	*leucophrys*	39.9403	-83.0325	1962.00	15.97414	4.15735	4.24847
BLB	7412	*Zonotrichia*	*leucophrys*	39.9833	-82.9167	1965.00	70.65351	10.21415	33.76398
BLB	8296	*Zonotrichia*	*leucophrys*	41.5333	-82.9333	1966.00	27.97853	0	0
BLB	8774	*Zonotrichia*	*leucophrys*	39.6667	-82.6667	1967.00	39.12465	8.50096	7.60048
BLB	9398	*Zonotrichia*	*leucophrys*	40.0833	-82.9167	1968.00	33.71038	0	4.10643
BLB	23900	*Zonotrichia*	*leucophrys*	58.7333	-93.8167	1997.00	15.90111	11.96955	3.7654
BLB	25372	*Zonotrichia*	*leucophrys*	40	-83	1999.00	0	0	7.76932
BLB	26743	*Zonotrichia*	*leucophrys*	40	-83	2001.00	9.91466	0	0
BLB	21132	*Zonotrichia*	*leucophrys*	39.6333	-123.7833	2003.00	35.5301	3.77545	15.35037

BLB	21133	*Zonotrichia*	*leucophrys*	39.6	-123.7833	2003.00	0	4.0602	4.11849
BLB	22428	*Zonotrichia*	*leucophrys*	39.3	-123.7833	2003.00	8.23631	0	0
BLB	22887	*Zonotrichia*	*leucophrys*	37.7289	-122.4948	1971.00	11.79334	0	3.94697
BLB	23083	*Zonotrichia*	*leucophrys*	39.0333	-123.7	2003.00	3.67696	3.03309	3.15436
BLB	23326	*Zonotrichia*	*leucophrys*	39	-123.6833	2003.00	15.26461	3.97645	3.58919
BLB	23537	*Zonotrichia*	*leucophrys*	39	-123.6833	2003.00	8.47148	0	0
BLB	23882	*Zonotrichia*	*leucophrys*	37.7289	-122.4948	1971.00	3.98315	7.68155	0
BLB	23941	*Zonotrichia*	*leucophrys*	36.75	-121.75	1997.00	3.87059	0	0
BLB	23948	*Zonotrichia*	*leucophrys*	36.7833	-121.75	1997.00	35.53948	0	0
BLB	24518	*Zonotrichia*	*leucophrys*	37.7289	-122.4948	1971.00	11.17627	0	0
BLB	24688	*Zonotrichia*	*leucophrys*	38.0833	-122.9167	1998.00	10.63089	6.15931	0
BLB	24705	*Zonotrichia*	*leucophrys*	38.0833	-122.9167	1998.00	20.23735	3.8592	0
BLB	24706	*Zonotrichia*	*leucophrys*	38.0833	-122.9167	1998.00	10.18668	0	0
BLB	24707	*Zonotrichia*	*leucophrys*	38.0833	-122.9167	1998.00	7.59914	3.83441	7.24538
BLB	24709	*Zonotrichia*	*leucophrys*	38.0833	-122.9167	1998.00	4.41731	0	0
BLB	24710	*Zonotrichia*	*leucophrys*	38.0833	-122.9167	1998.00	36.64565	0	7.93548
BLB	24711	*Zonotrichia*	*leucophrys*	38.0833	-122.9167	1998.00	3.38953	0	0
BLB	24713	*Zonotrichia*	*leucophrys*	38.0833	-122.9167	1998.00	19.58477	5.48328	0
BLB	24714	*Zonotrichia*	*leucophrys*	38.0833	-122.9167	1998.00	3.50477	0	0
BLB	26976	*Zonotrichia*	*leucophrys*	37.8569	-122.2978	1971.00	4.20291	4.04144	0
BLB	28219	*Zonotrichia*	*leucophrys*	38.5667	-123.3333	2003.00	4.21631	0	8.52508
BLB	29612	*Zonotrichia*	*leucophrys*	38.3022	-123.0572	1993.00	8.27651	3.99454	0
BLB	32507	*Zonotrichia*	*leucophrys*	37.6139	-122.4869	1981.00	7.43633	0	0
BLB	33184	*Zonotrichia*	*leucophrys*	37.7667	-122.45	1981.00	24.24462	7.08927	9.45102
BLB	33185	*Zonotrichia*	*leucophrys*	37.7667	-122.45	1981.00	56.16275	3.1088	0
BLB	33261	*Zonotrichia*	*leucophrys*	37.9667	-119.1	1982.00	4.17477	0	0
BLB	33332	*Zonotrichia*	*leucophrys*	37.7289	-122.4948	1974.00	16.01032	3.55368	10.42118
BLB	33407	*Zonotrichia*	*leucophrys*	37.7667	-122.4667	1981.00	3.96238	0	0
BLB	33463	*Zonotrichia*	*leucophrys*	37.7125	-122.4992	1990.00	21.40717	4.00124	4.00124
BLB	33665	*Zonotrichia*	*leucophrys*	37.7289	-122.4948	1969.00	20.71774	0	0
BLB	33861	*Zonotrichia*	*leucophrys*	38.0667	-122.8844	1985.00	7.68155	0	13.7819
BLB	33944	*Zonotrichia*	*leucophrys*	37.7667	-122.45	1982.00	10.69588	3.95501	3.95501
BLB	34043	*Zonotrichia*	*leucophrys*	37.7669	-122.4894	1991.00	3.15704	3.1624	0
BLB	34281	*Zonotrichia*	*leucophrys*	37.803	-122.4745	1992.00	19.25714	0	7.81488
BLB	34417	*Zonotrichia*	*leucophrys*	37.7667	-122.4667	1993.00	17.84612	0	0
BLB	34570	*Zonotrichia*	*leucophrys*	37.7667	-122.4667	1992.00	15.18019	0	10.95383
BLB	34823	*Zonotrichia*	*leucophrys*	36.5217	-121.9528	1976.00	17.6813	0	0
BLB	35222	*Zonotrichia*	*leucophrys*	37.7667	-122.4667	1968.00	13.06165	0	6.64908
BLB	35290	*Zonotrichia*	*leucophrys*	37.7667	-122.4667	1969.00	9.75855	0	0

BLB	35490	*Zonotrichia*	*leucophrys*	32.5839	-117.1131	1968.00	3.93625	0	0
BLB	35513	*Zonotrichia*	*leucophrys*	37.93	-122.7353	1969.00	6.83132	0	0
BLB	35517	*Zonotrichia*	*leucophrys*	37.3192	-122.2742	1969.00	0	0	6.22564
BLB	35525	*Zonotrichia*	*leucophrys*	37.9058	-122.2386	1969.00	6.36701	0	0
BLB	35732	*Zonotrichia*	*leucophrys*	37.8	-122.4636	1969.00	0	2.99557	3.09674
BLB	35795	*Zonotrichia*	*leucophrys*	37.9158	-122.3117	1970.00	10.16658	0	0
BLB	35806	*Zonotrichia*	*leucophrys*	38.041	-122.7994	1970.00	0	0	0
BLB	35896	*Zonotrichia*	*leucophrys*	37.7289	-122.4948	1970.00	17.96471	0	0
BLB	35955	*Zonotrichia*	*leucophrys*	37.7667	-122.45	1971.00	2.57816	0	0
BLB	36059	*Zonotrichia*	*leucophrys*	37.7667	-122.4667	1971.00	12.84725	4.48498	4.48498
BLB	36060	*Zonotrichia*	*leucophrys*	37.7667	-122.4667	1971.00	15.72423	0	15.84416
BLB	36166	*Zonotrichia*	*leucophrys*	37.8569	-122.2978	1971.00	12.04459	0	0
BLB	37006	*Zonotrichia*	*leucophrys*	35.068	-120.6093	1982.00	18.72114	0	0
BLB	38164	*Zonotrichia*	*leucophrys*	37.874	-122.239	1970.00	3.90409	3.8793	7.38072
BLB	39486	*Zonotrichia*	*leucophrys*	37.7667	-122.4667	1995.00	42.66962	10.96254	3.74999
BLB	39997	*Zonotrichia*	*leucophrys*	38.3174	-123.0711	1995.00	49.03261	0	0
BLB	41913	*Zonotrichia*	*leucophrys*	37.7667	-122.4667	1994.00	7.27687	4.13993	3.67294
BLB	42360	*Zonotrichia*	*leucophrys*	37.7883	-122.4608	1977.00	8.24301	0	8.02392
BLB	42500	*Zonotrichia*	*leucophrys*	37.7667	-122.4667	1992.00	47.73147	3.7453	3.68232
XC	445848	*Zonotrichia*	*leucophrys*	39.3081	-123.81	2015.00	11.77592	3.69974	0
XC	445849	*Zonotrichia*	*leucophrys*	39.2263	-123.7203	2015.00	24.39135	4.13658	8.22894
XC	459465	*Zonotrichia*	*leucophrys*	37.771	-122.4924	2019.00	11.66336	3.79354	0
XC	610641	*Zonotrichia*	*leucophrys*	37.9085	-122.3511	2020.00	10.1773	0	3.57579
BLB	6596	*Zonotrichia*	*leucophrys*	41.3333	-106.3	1963.00	11.03892	0	3.89203
BLB	6597	*Zonotrichia*	*leucophrys*	43.0167	-110.1167	1963.00	36.11233	4.05283	0
BLB	20614	*Zonotrichia*	*leucophrys*	38.3333	-119.6333	1993.00	6.38711	0	0
BLB	20616	*Zonotrichia*	*leucophrys*	38.3333	-119.6333	1993.00	37.4597	10.85802	29.12691
BLB	20617	*Zonotrichia*	*leucophrys*	38.3333	-119.6333	1993.00	92.85597	4.07092	4.27929
BLB	20628	*Zonotrichia*	*leucophrys*	38.7	-119.9833	1993.00	118.01246	14.81236	15.68336
BLB	32036	*Zonotrichia*	*leucophrys*	37.9	-119.1167	1980.00	10.81581	0	0
BLB	33278	*Zonotrichia*	*leucophrys*	37.9	-119.1167	1984.00	23.70192	0	0
BLB	33894	*Zonotrichia*	*leucophrys*	37.9778	-119.1019	1988.00	3.84513	6.00387	10.84462
BLB	33967	*Zonotrichia*	*leucophrys*	42.4167	-119.6667	1975.00	7.76195	0	0
BLB	35008	*Zonotrichia*	*leucophrys*	37.9667	-119.1	1989.00	7.46045	0	10.41448
BLB	7718	*Zonotrichia*	*leucophrys*	44.5833	-123.3167	1965.00	55.007	7.64403	7.74118
BLB	24672	*Zonotrichia*	*leucophrys*	40	-83	1998.00	3.86925	0	4.05216
BLB	24739	*Zonotrichia*	*leucophrys*	46.25	-124.0833	1998.00	99.75094	8.13849	29.3192
BLB	24773	*Zonotrichia*	*leucophrys*	46.25	-124.0833	1998.00	2.82405	0	0
BLB	24869	*Zonotrichia*	*leucophrys*	46.2167	-124	1998.00	11.6848	0	0

BLB	24878	*Zonotrichia*	*leucophrys*	46.25	-124.0833	1998.00	11.62919	3.61465	0
BLB	24880	*Zonotrichia*	*leucophrys*	46.25	-124.0833	1998.00	19.42531	3.90476	0
BLB	24881	*Zonotrichia*	*leucophrys*	46.2286	-124.01	1998.00	3.44715	3.64212	0
BLB	24882	*Zonotrichia*	*leucophrys*	46.2167	-124	1998.00	60.43936	8.19477	32.07826
BLB	24883	*Zonotrichia*	*leucophrys*	46.2167	-124	1998.00	123.72957	20.11541	22.4785
BLB	24894	*Zonotrichia*	*leucophrys*	46.2286	-124.01	1998.00	76.87044	12.1672	41.55943
BLB	25949	*Zonotrichia*	*leucophrys*	48.5	-124.5	1999.00	50.6989	3.98516	11.76989
BLB	26285	*Zonotrichia*	*leucophrys*	48.2	-122.7	2000.00	13.8623	4.42468	0
BLB	26355	*Zonotrichia*	*leucophrys*	46.2847	-124.0769	2000.00	7.80483	3.71984	0
BLB	26845	*Zonotrichia*	*leucophrys*	48	-123.75	2001.00	12.13303	4.27259	0
BLB	29176	*Zonotrichia*	*leucophrys*	40.4833	-124.1	2004.00	10.99068	0	0
BLB	29177	*Zonotrichia*	*leucophrys*	41.75	-124.2	2004.00	4.00325	0	3.5309
BLB	31650	*Zonotrichia*	*leucophrys*	41.9283	-124.1458	2005.00	0	3.7654	3.60862
BLB	33235	*Zonotrichia*	*leucophrys*	37.7667	-122.45	1980.00	23.87277	2.04082	0
BLB	33834	*Zonotrichia*	*leucophrys*	47.0072	-122.9094	1977.00	8.36562	0	3.90744
BLB	35011	*Zonotrichia*	*leucophrys*	43.9667	-124.0833	1985.00	46.79682	0	13.45896
BLB	35043	*Zonotrichia*	*leucophrys*	34.4	-119.8333	1968.00	10.8875	0	7.96362
BLB	35047	*Zonotrichia*	*leucophrys*	34.4	-119.8333	1968.00	3.9061	3.22404	0
BLB	35110	*Zonotrichia*	*leucophrys*	34.4631	-119.8319	1968.00	0	3.92285	3.94831
BLB	35123	*Zonotrichia*	*leucophrys*	34.4	-119.8333	1968.00	11.61847	3.95099	0
BLB	35124	*Zonotrichia*	*leucophrys*	34.4	-119.8333	1969.00	10.47679	0	0
BLB	35144	*Zonotrichia*	*leucophrys*	34.4	-119.8333	1970.00	6.82663	3.47931	3.47931
BLB	36565	*Zonotrichia*	*leucophrys*	40.7891	-124.176	1970.00	24.74846	0	0
BLB	36712	*Zonotrichia*	*leucophrys*	43.0667	-124.4333	1970.00	13.80133	0	0
BLB	36819	*Zonotrichia*	*leucophrys*	44.6378	-124.0339	1970.00	11.58564	0	3.57244
BLB	36820	*Zonotrichia*	*leucophrys*	45.4627	-123.8366	1970.00	17.43273	3.63341	0
BLB	36893	*Zonotrichia*	*leucophrys*	46.25	-124.0833	1970.00	0	0	3.83374
BLB	36961	*Zonotrichia*	*leucophrys*	34.4124	-119.8518	1982.00	29.71182	3.6582	10.18199
BLB	37080	*Zonotrichia*	*leucophrys*	47.5507	-122.0442	1970.00	11.10458	3.82168	7.68356
BLB	37085	*Zonotrichia*	*leucophrys*	47.3045	-122.5201	1970.00	11.74644	0	3.73458
BLB	37704	*Zonotrichia*	*leucophrys*	46.707	-123.98	1970.00	16.5088	0	16.57848
BLB	38159	*Zonotrichia*	*leucophrys*	42.8411	-124.5419	1970.00	11.86034	0	7.64872
BLB	38431	*Zonotrichia*	*leucophrys*	42.7329	-119.5507	1977.00	8.04	0	0
BLB	39290	*Zonotrichia*	*leucophrys*	34.4243	-119.7511	1986.00	4.76772	0	0
BLB	39650	*Zonotrichia*	*leucophrys*	36.9664	-95.844	1983.00	2.9346	0	0
BLB	40686	*Zonotrichia*	*leucophrys*	48.7376	-122.7929	1977.00	31.35466	0	16.44314
BLB	42570	*Zonotrichia*	*leucophrys*	34.4631	-119.8319	1967.00	3.56172	0	6.499
XC	160205	*Zonotrichia*	*leucophrys*	48.395338	-123.599262	2013.00	7.70165	0	0
XC	182564	*Zonotrichia*	*leucophrys*	48.5093	-123.4537	2014.00	0	0	4.17075

XC	253833	*Zonotrichia*	*leucophrys*	44.3799	-121.6917	2015.00	7.50199	0	3.14096
XC	253837	*Zonotrichia*	*leucophrys*	43.661	-124.207	2015.00	7.9864	3.39623	0
XC	253839	*Zonotrichia*	*leucophrys*	44.753	-123.3028	2015.00	7.035	0	0
XC	570694	*Zonotrichia*	*leucophrys*	48.4269	-123.3797	2020.00	3.79354	3.69237	0
BLB	218	*Zonotrichia*	*leucophrys*	39.9961	-83.0189	1951.00	18.97038	0	0
BLB	1378	*Zonotrichia*	*leucophrys*	39.6667	-82.6667	1955.00	41.55809	8.49024	0
BLB	11529	*Zonotrichia*	*leucophrys*	39.3264	-119.8778	1971.00	11.8389	0	8.13581
BLB	11538	*Zonotrichia*	*leucophrys*	36.6167	-121.8167	1971.00	23.55251	3.81431	4.13457
BLB	11543	*Zonotrichia*	*leucophrys*	36.5	-121.8333	1971.00	31.85515	5.71778	0
BLB	11545	*Zonotrichia*	*leucophrys*	36.5	-121.8333	1971.00	3.75334	0	8.14586
BLB	12866	*Zonotrichia*	*leucophrys*	40.0833	-82.9167	1974.00	8.33614	0	0
BLB	14778	*Zonotrichia*	*leucophrys*	41.5333	-82.9333	1978.00	15.50246	0	4.10978
BLB	18009	*Zonotrichia*	*leucophrys*	58.725	-94.1167	1989.00	15.66192	0	0
BLB	18605	*Zonotrichia*	*leucophrys*	61.5294	-149.9075	1993.00	62.91501	4.21564	10.66439
BLB	18630	*Zonotrichia*	*leucophrys*	63.3333	-150.5006	1993.00	4.1674	4.12184	0
BLB	18749	*Zonotrichia*	*leucophrys*	39.5833	-120.4833	1992.00	20.00486	0	0
BLB	20613	*Zonotrichia*	*leucophrys*	38.3333	-119.6333	1993.00	3.90811	3.93558	0
BLB	21131	*Zonotrichia*	*leucophrys*	39.6333	-123.7833	2003.00	13.28141	0	0
BLB	23093	*Zonotrichia*	*leucophrys*	58.725	-94.1167	1959.00	16.51885	4.17075	4.16539
BLB	23094	*Zonotrichia*	*leucophrys*	51.1667	-80.3333	1958.00	29.55705	0	0
BLB	23681	*Zonotrichia*	*leucophrys*	37.9	-119.1167	1990.00	27.52025	3.63475	0
BLB	23863	*Zonotrichia*	*leucophrys*	58.7333	-93.8167	1997.00	19.83401	0	0
BLB	23874	*Zonotrichia*	*leucophrys*	38.5	-121.75	1997.00	50.90928	9.21317	10.93507
BLB	23901	*Zonotrichia*	*leucophrys*	58.7333	-93.8167	1997.00	19.91374	4.24981	0
BLB	23902	*Zonotrichia*	*leucophrys*	58.7333	-93.8167	1997.00	27.02847	0	4.10643
BLB	23928	*Zonotrichia*	*leucophrys*	38.5	-121.75	1991.00	49.57933	3.53023	2.34433
BLB	23939	*Zonotrichia*	*leucophrys*	36.75	-121.75	1997.00	6.40654	0	3.417
BLB	24673	*Zonotrichia*	*leucophrys*	38.5	-121.75	1998.00	39.74976	3.66624	0
BLB	24692	*Zonotrichia*	*leucophrys*	40	-83	1998.00	11.46571	0	0
BLB	24738	*Zonotrichia*	*leucophrys*	46.2286	-124.01	1998.00	14.82174	4.69402	2.16812
BLB	27439	*Zonotrichia*	*leucophrys*	38.7	-119.9833	1995.00	12.91157	0	3.6582
BLB	30928	*Zonotrichia*	*leucophrys*	48.15	-123.2	1996.00	10.10561	0	0
BLB	34996	*Zonotrichia*	*leucophrys*	39.6833	-120.4	1989.00	10.69454	0	3.47931
BLB	35122	*Zonotrichia*	*leucophrys*	?	?	1968.00	9.50931	0	0
BLB	35139	*Zonotrichia*	*leucophrys*	37.6	-112.8333	1970.00	6.70134	0	0
BLB	35142	*Zonotrichia*	*leucophrys*	39.6456	-111.2603	1970.00	7.38072	0	5.37675
BLB	35143	*Zonotrichia*	*leucophrys*	48.6833	-113.7167	1970.00	11.58095	4.1942	0
BLB	36217	*Zonotrichia*	*leucophrys*	37.8994	-119.2211	1975.00	10.79638	0	0
BLB	37497	*Zonotrichia*	*leucophrys*	42.0656	-124.3083	1977.00	11.61981	0	3.93491

BLB	37511	*Zonotrichia*	*leucophrys*	42.3888	-122.3306	1977.00	1.97851	0	0
BLB	37792	*Zonotrichia*	*leucophrys*	34.4095	-119.7471	1984.00	0	0	0
BLB	38950	*Zonotrichia*	*leucophrys*	37.7698	-122.4658	1984.00	14.16514	0	0
BLB	39343	*Zonotrichia*	*leucophrys*	37.9465	-119.2063	1992.00	16.19591	0	0
BLB	41320	*Zonotrichia*	*leucophrys*	45.2003	-117.2786	1977.00	20.5422	0	0
BLB	41369	*Zonotrichia*	*leucophrys*	34.1006	-116.8278	1977.00	27.78624	8.2946	4.34696
BLB	42503	*Zonotrichia*	*leucophrys*	37.8667	-122.3	1992.00	3.97846	4.3483	0
BLB	42505	*Zonotrichia*	*leucophrys*	37.8667	-122.3	1992.00	34.72677	0	4.37242
BLB	42835	*Zonotrichia*	*leucophrys*	?	?	?	0	0	4.11112

* Individuals marked with asterisks were mis-identified as *Cardinalis sinuatus* in the databases and are now correctly identified as *Cardinalis cardinalis*.
