# Supplementary material for: The impacts of fine-tuning, phylogenetic distance, and sample size on big-data bioacoustics
Source: PLoS One. 2022 Dec 7;17(12):e0278522. doi: 10.1371/journal.pone.0278522 (PMC9728902; doi:10.1371/journal.pone.0278522)
Supplement: Attachment — Submitted filename: ProvostYangCarstens_Revision_Response.pdf [file pone.0278522.s002.pdf]

1. Thank you very much for all of your comments. In this letter we have set off our responses in a different font as well as color, and they are also marked with numbers before each response.

PONE-D-22-07358
The impacts of transfer learning, phylogenetic distance, and sample size on big-data bioacoustics
PLOS ONE

Dear Dr. Provost,

Thank you for submitting your manuscript to PLOS ONE. After careful consideration, we feel that it has merit but does not fully meet PLOS ONE's publication criteria as it currently stands. Therefore, we invite you to submit a revised version of the manuscript that addresses the points raised during the review process.

Please include the following items when submitting your revised manuscript:

A rebuttal letter that responds to each point raised by the academic editor and reviewer(s). You should upload this letter as a separate file labeled 'Response to Reviewers'.

A marked-up copy of your manuscript that highlights changes made to the original version. You should upload this as a separate file labeled 'Revised Manuscript with Track Changes'.

An unmarked version of your revised paper without tracked changes. You should upload this as a separate file labeled 'Manuscript'.

1. Please ensure that your manuscript meets PLOS ONE's style requirements, including those for file naming. The PLOS ONE style templates can be found at

https://journals.plos.org/plosone/s/file?id=wjVg/PLOSOne_formatting_sample_main_body.pdf
and

https://journals.plos.org/plosone/s/file?id=ba62/PLOSOne_formatting_sample_title_authors_affiliations.pdf

2. Thank you for stating the following in the Acknowledgments Section of your manuscript:

"KLP and BCC were supported by NSF DEB-2016189."

We note that you have provided additional information within the Acknowledgements Section that is not currently declared in your Funding Statement. Please note that funding information should not appear in the Acknowledgments section or other areas of your manuscript. We will

only publish funding information present in the Funding Statement section of the online submission form.

Please remove any funding-related text from the manuscript and let us know how you would like to update your Funding Statement. Currently, your Funding Statement reads as follows:

"KLP and BCC were supported by the National Science Foundation DEB-2016189 [https://www.nsf.gov/awardsearch/showAward?AWD_ID=2016189]. The funders had no role in study design, data collection and analysis, decision to publish, or preparation of the manuscript."

Please include your amended statements within your cover letter; we will change the online submission form on your behalf.

3. In your Data Availability statement, you have not specified where the minimal data set underlying the results described in your manuscript can be found. PLOS defines a study's minimal data set as the underlying data used to reach the conclusions drawn in the manuscript and any additional data required to replicate the reported study findings in their entirety. All PLOS journals require that the minimal data set be made fully available. For more information about our data policy, please see http://journals.plos.org/plosone/s/data-availability.

Upon re-submitting your revised manuscript, please upload your study's minimal underlying data set as either Supporting Information files or to a stable, public repository and include the relevant URLs, DOIs, or accession numbers within your revised cover letter. For a list of acceptable repositories, please see http://journals.plos.org/plosone/s/data-availability#loc-recommended-repositories. Any potentially identifying patient information must be fully anonymized.

We will update your Data Availability statement to reflect the information you provide in your cover letter.

4. We note that you have stated that you will provide repository information for your data at acceptance. Should your manuscript be accepted for publication, we will hold it until you provide the relevant accession numbers or DOIs necessary to access your data. If you wish to make changes to your Data Availability statement, please describe these changes in your cover letter and we will update your Data Availability statement to reflect the information you provide.

5. Please include captions for your Supporting Information files at the end of your manuscript, and update any in-text citations to match accordingly. Please see our Supporting Information guidelines for more information: http://journals.plos.org/plosone/s/supporting-information

[Note: HTML markup is below. Please do not edit.]

Reviewers' comments:

Reviewer #1: This paper analyzed the impact of various factors including transfer learning, phylogenetic distance and sample size on classifying the birds with their vocalizations and machine learning techniques.

2. Thank you very much for your comments!

Major comments:

1. This paper provided detailed explanation about data preparation and the model training setup, but the major concern is that it is not clear what questions this paper is trying to answers, or gaps to fill or how to generalize the learning from this paper. This paper started with introduction of the researches of vocalizations in birds and then the introduction (and some discussion) of machine learning models, like classic machine learning vs deep learning vs transfer learning. However, it did not mention why there is such a need. It is not clear where the research gap is and how the learning/discussion in this paper can be generalized. For example, if this paper aims to improve the state of art accuracy, it should be clearly stated in the introduction and also cite the existing works. Or if this paper aims to guide researchers in the bioacoustics area to fine tune the existing ML models, the authors should clearly state and clarify why the researcher in this area need such guideline

3. Thank you for pointing out this clear omission. We worked to clarify the text in the entire manuscript to make it clearer both to experts and non-experts why this work is needed and how it points the way to future research.

2. The Introduction section introduced multiple concepts: vocalization, data richness, heavy human labeling work, machine learning, deep learning, transfer learning, but they are unconnected dots and did not lead to clear research questions. It is also not clear about the reason to introduce each topic/concept.

4. We have clarified this and added better transition sentences between the sections.

3. If TweetyNet is the main model to be tuned in this paper, the authors should introduce this model more and discuss about its performance, strength and weakness.

5. We have discussed pros and cons of using TweetyNet. Some of this discussion is in the TweetyNet paper itself and so we summarize that.

4. The annotations/labels accuracy and consistency should be measured when they are used as label to train model.

6. We have added more information about model performance to address this concern, including accuracy, recall, precision, and F-score measures as well as segment error rate.

5. The Song data summary should be listed in the Method instead of Results since this is not the main purpose/result of this paper. It can also help audience to understand the data size while reading the method

7. We have moved this section into the methods.

6. This paper selected 6 species where the data is richly available to test the impact, please clarify why the learning from the 6 species can be generalized to all the species

8. We added more species that were likewise distributed across the tree. These species do not have as many recordings available as the original selection but they overlap more closely with the complexity dataset. We now have a total of 9 species, where all but 3 do not have data from the Medina and Francis 2012 manuscript. We have also shown that the correlation between complexity and performance is still there. We feel confident to say that in Passeriform birds (~70% of extant species) our method will work.

7. It is commonly known that balanced dataset should have a better performance than unbalanced dataset, please clarify why the authors may think the unbalanced dataset may be better?

9. We have omitted the unbalanced dataset from the final manuscript.

8. Table 2 is the accuracy of the models, it looks like a recall rate (i.e., correct prediction / ground truth). Please also provide the precision (correct prediction / all predictions for each species) for a more comprehensive assessment of the model.

10. We have calculated precision, recall, and f-score for each of the models.

In-line comments;

1. Line 69, "where the features used do not need to be pre-trained". The word "pre-trained" may not be accurate, the word "prepared" could be better

11. We have changed this.

2. Line 96, in the transfer learning, the pre-trained ANNs should not be directly applied to new, related data. Some additional training should be needed

12. We realized after these revisions that we were using "transfer learning" incorrectly in this context. What we want to know is whether models trained on one species can be applied to another without additional training. We have updated the text to reflect this.

3. Line 112, the Github link does not work

13. We had accidentally set the GitHub link to be a private repository. We have fixed this.

4. Line 152-155, this information should be in Introduction or mentioned at the beginning of the Method

14. Fixed

5. Line 180-184, please provide references or rational for the parameter selection

15. Fixed

6. Line 194, which is the reason for choosing 5-step and 50-step checkpoints? Would more checkpoints setting provide a better understanding about model performance change with the number of steps change?

16. We have discussed this method with the TweetyNet developers who instead informed us we needed to proceed for longer checkpoints (100) and also change our parameter space. Originally we wanted to include this to verify whether training models for longer would

cause them to perform better -- it does. However we do not think that this is relevant anymore and as such we have omitted all comparisons between checkpoints.

Reviewer #2: This paper explored the consequences of model training decisions on the accuracy of an avian classification algorithm. The authors adopted a recently published neural network architecture which links convolutional and recurrent neural networks to predict both time-specific song segmentation boundaries (onset time and call end time) and song labels (colloquially predicting the species that made a specific call) and used this model architecture with an unbalanced (intentionally) constructed avian audio dataset to evaluate the effects of model training decisions on classification accuracy. The primary contribution of this manuscript is not the development of a new avian audio classification algorithm, but to provide practical model training advice and attempt to estimate some of the factors influencing the outcomes of model training decisions. There are substantial knowledge gaps in this field. Specifically, advances in structural or conceptual model development are far outpacing studies evaluating best practices and exploration of the factors influencing the 'predictability' of certain call types.

17. Thank you Matthew Weldy for your comments!

1. The study presents the results of original research.
This study presents the results of original research, and contributes to a substantial and growing knowledge gap. Advances in computer vision and hearing algorithms (and the overlap in between) and their application to ecological problems are far outpacing rigorous studies establishing best practices and evaluating the consequences of training decisions.

2. Results reported have not been published elsewhere.
To the extent of my knowledge the work presented here has not been published elsewhere. The model architecture used as a case study has been published, but the training evaluations and call complexity analyses have not been reported elsewhere.

3. Experiments, statistics, and other analyses are performed to a high technical standard and are described in sufficient detail.

The analytical framework and techniques used by the authors was reasonable. However, I have a few concerns.

A) Throughout the manuscript the authors refer to evaluations of accuracy; however, it is only inferred through context that they are only referring to classification accuracy. The TweetyNet algorithm makes two types of predictions (i.e. similar to YOLO or similar models which predict bounding boxes and classes) and both predictions can be evaluated for accuracy. The authors appear primarily concerned with an assessment of clip-level classification accuracy, which on its own makes a valuable contribution. Please make this decision explicit throughout the manuscript. But, an evaluation of temporal segmentation accuracy would extend the influence and usefulness of this evaluation in regards to establishing semi-automated labeling techniques.

18. We were specifically looking at clip level classification accuracy, yes, but we have also added syllable error rate which is an assessment of segmentation accuracy into the analysis.

B) I understand that the number of samples used for some of the species was an attempt to explore the consequences of sparsely represented classes. But, for all but two of the species, the sample sizes are well below those that would be used to develop a classification algorithm for applied projects.

19. Unfortunately, this reflects the reality of the data we are seeking to train on more broadly. Most bird species do not have sufficient sample sizes to classify their syllables alone. However, we are not looking to use a classification algorithm that identifies the species -- rather, we are trying to identify the presence (as well as onset and offset) of syllables. All birds that vocalize have syllables although they are variable between species. Further, we chose TweetyNet in particular because its underlying cNN+rNN framework was shown to be highly accurate with these limited datasets. Our goal for this manuscript is to create use-cases for later researchers such that they do not spend their precious research time parameterizing models and annotating data.

C) I have some concern that there is a slight mismatch in the use of the term ‘transfer learning.’ Technically, the authors approach is a transfer learning approach (using the weights of pretrained models. However, there is a big mismatch between the general usage of the term and the application presented here. For example, transfer learning approaches are often used to harness the power of large-scale models trained across generalized datasets, which are computationally inefficient to re-run or require impractical amounts of computing power. The general thought is that the weights learned in these large-scale models are able to capture general patterns in visual or audio data and that by fine tuning small layers on top of these base weights it will be easier and faster to develop specific solutions. I have concerns that the findings presented here would not generalize to broader applications of transfer learning.

20. Thank you for this comment. Biological data are unique in the sense that there is shared history across the units of analysis. In this case, we analyze data from different species, yet these data are not statistically independent due to common ancestry (i.e., their phylogenetic history). For this reason it isn’t possible to create a ‘generalized’ data set of bird song recordings because there are hidden correlations among the individual calls due to this ancestry. We anticipate that these findings (i.e., particularly that the utility of a model trained using data from species ‘A’ would be applicable to species ‘B’ would have broad application to biological data in a manner that is proportional to their phylogenetic distance), but of course have not conducted this analysis.

D) The authors performed a principal components analysis on the output of the SoundShape pipeline, which I assume was performed to reduce the dimensionality of the R package output. The authors then stated that the ‘used the three PCs to calculate a hypervolume per species.’ In general, PCA can be sensitive to arbitrary analytical decisions and I would feel more comfortable interpreting the results if I could see the scree-plot of the Eigenvectors or if the authors had clearly stated a selection rule. As it currently stands, this component of the analysis is not reproducible. Some other small points associated with this are that the output of a PCA can differ between packages (often due to differences in how the packages handle missing values) and the authors did not document the package, and that the irregularities presented in

the results section (lines 388- 397) might be explained by the cutoff used in selecting the number of PCs to retain, particularly if the variation described by lower-level Eigenvectors was only present in the data rich species.

21. This is a good critique. To address this we set a cutoff of 50% accuracy. We originally were doing this with the first 3 PCs due to memory constraints with the convex hull algorithm for calculating hypervolumes. However we have changed our analysis to alleviate those constraints.

E) I found the hypervolume and song complexity analysis useful in thinking about the differences in classification performance; however, I feel that careful selection of study species could have increased the utility of this analysis. For example, if the authors had selected more species from the song complexity analysis [68] so they could have estimated correlation coefficients, or recreated the analysis performed in [68] so they could estimate correlations using the data on hand. I understand that the authors selected the 6 species used here because, 'they were distributed across the phylogenetic tree and had relatively high numbers of recordings available,' which had implications for the phylogenetic similarity analysis. However, the two conditions described above could have been satisfied by a large number of species, including a number of other species included in [68].

22. We added more species from this complexity analysis to our dataset here. Our results do not change appreciably -- more complex species are harder to optimize.

F) The regression analysis described on lines 252-266 is not reproducible in its current format, nor do the authors provide enough output to assess the analysis. For example, the authors state they used generalized linear regressions, ANOVA, and Tukey's HSD tests. However, the following paragraph only discusses linear regressions (not GLMs), it is unclear if the authors fit independent univariate models or a single multivariate model (if the latter are the predictors correlated?), and there is not enough information presented to determine if the Tukey's HSD test was employed across all comparisons as a multiple test correction or if a multiple correction was used. Lastly the regression coefficients are not presented in the analysis.

23. We clarified this in the text. Further we have put a full list of the models used and all of their associated coefficients into the supplement.

G) Briefly, some of the accuracy comparisons would have benefitted by developing bootstrapped confidence intervals. Doing so would provide more information to interpret if differences in accuracy were real or reasonable stochastic variability.

24. We now also report mean and standard deviations of accuracy and other performance metrics along with our point estimates.

4. Conclusions are presented in an appropriate fashion and are supported by the data.
Overall, the conclusions are appropriate and there are a couple key points which make valuable contributions to the field. In particular, I found the recommendations on lines 463-469 pragmatic and useful.

25. Thank you!

I do think the paper would be stronger with some expanded discussion about the analyses and their applications. For example, there was very limited discussion about the limitations of the call song complexity and hypervolume analysis or the ecological or phylogenetic implications of this analysis. Perhaps the limited scope of this analytical component could be effectively supplemented with a visual representation of the different songs. I would expect calls with similar hypervolumes to appear visually similar (spanning long frequency or temporal windows).

26. As you can see in the sonograms in Figure 2, this is true in some cases but not all. We have improved discussion of this figure in an effort to better make this point.

I would prefer additional detail in some sections, but to some extent this is stylistic. I do caution the authors to keep the discussion and conclusion sections focused on their contributions and not on the contributions made by the authors of the TweetyNet algorithm. For example, lines 488-490 claim that the authors “application can supplement the deep avian bioacoustics literature with the breadth afforded by efficient data generation.” I would argue that these contributions and the application were made by the authors of TweetyNet and that the authors of this manuscript did not develop an application or new processing algorithm that reduces the burden of efficient data generation (i.e. labeling).

27. Thank you for this comment. We have attempted to focus our discussion on the contributions of this work rather than also including aspects of TweetyNet.

In addition, I felt the discussion about clustering-based classification (lines 514-518) outside the scope of the analysis.

28. This has been removed. Clustering in this case is particularly important when moving from segmentation to syntax, but that’s beyond the scope of this manuscript at present.

5. The article is presented in an intelligible fashion and is written in standard English.
The manuscript is presented in standard English. There are a number of confusing run-on sentences, including a paragraph consisting of a single 4-clause sentence near the end of the introduction. In addition, there are a few places where I feel (and in full disclosure that this is opinion, so the authors can address as they see fit) the authors chose concise or loose sentence structures at the expense of clarity. For example, on lines 291-292 it is unclear that the highlighted findings are in reference to the transfer learning conditions.
Broadly speaking, a careful attempt to reduce jargon throughout the paper and clearly define terms when they are standard or necessary (“zero-shot”, “few-shot”) would make the paper more accessible.
One specific spot I would recommend revisiting would be the first paragraph of the Machine Learning section in the Introduction. In particular the second half of the paragraph was not informative (e.g. what is a slew of problems, why is that slew important, are there specific tensions between these problems and ecological data types). Also, at least 2 of the three machine learning algorithms listed have deep roots in probabilistic traditions (linear regression, and MaxEnt (as a Poisson process). Although, both have been adopted to ‘learning’ inferential paradigms.

29. Thank you for highlighting this. We have gone through the manuscript carefully with an eye towards improving readability and revising the writing to be more concise and direct.

6. The research meets all applicable standards for the ethics of experimentation and research integrity.

This paper does not violate any ethics of experimentation. However, I have some concerns from an analytical perspective that the authors performed a number of statistical tests (multiple regressions), did not use a multiple test correction (Bonferroni etc…), and only presented a p-value based summary for some of the tests instead of magnitudes and confidence intervals.

30. We have used a Bonferroni correction -- thank you for the suggestion.

7. The article adheres to appropriate reporting guidelines and community standards for data availability.

The authors claim that the data is available without restrictions. I was able to verify access to some of the files on the Borror Lab of Bioacoustics database and Xeno-Canto (I did not test access to all the files). The code repository is not currently accessible so I am unable to verify.

31. We accidentally set the code repository to private instead of unlisted. It is available now.
